# Supplementary material for: Is trust a zero-sum game? What happens when institutional sources get it wrong
Source: PLoS One. 2025 Apr 22;20(4):e0321743. doi: 10.1371/journal.pone.0321743 (PMC12013948; doi:10.1371/journal.pone.0321743)
Supplement: S1 File — (DOCX) [file pone.0321743.s001.docx]

**Is Trust a Zero-Sum Game — Supporting Information**

The current document reports alternative and additional analyses that are not included in the main manuscript.

**Table of** **Contents**

[Divergences from preregistration 4](#_Toc180668948)

[Manipulation Materials 5](#_Toc180668949)

[Table S1 6](#_Toc180668950)

[Table S2 8](#_Toc180668951)

[Excluding Participants Based on Memory Checks 11](#_Toc180668952)

[Table S3 12](#_Toc180668953)

[Table S4 13](#_Toc180668954)

[Table S5 14](#_Toc180668955)

[Main Analyses — Univariate ANOVAs 15](#_Toc180668956)

[Table S6 16](#_Toc180668957)

[Table S7 17](#_Toc180668958)

[Likelihood of Using Source (Single Item Analyses) 18](#_Toc180668959)

[Table S8 19](#_Toc180668960)

[Table S9 20](#_Toc180668961)

[Table S10 22](#_Toc180668962)

[Multiple Comparisons 24](#_Toc180668963)

[Table S11. 24](#_Toc180668964)

[Table S12 24](#_Toc180668965)

[Table S13 25](#_Toc180668966)

[Table S14 26](#_Toc180668967)

[Table S15 26](#_Toc180668968)

[Table S16 28](#_Toc180668969)

[Table S17 28](#_Toc180668970)

[Figure S1 29](#_Toc180668971)

[Additional Sources 30](#_Toc180668972)

[Table S18 31](#_Toc180668973)

[Correlations Split by Political Party 35](#_Toc180668974)

[Table S19 36](#_Toc180668975)

[Table S20 37](#_Toc180668976)

[Table S21 38](#_Toc180668977)

# Divergences from preregistration

All three studies were preregistered, and the registrations can be viewed on OSF (<https://osf.io/hms3q/>). Here we report several divergences from the preregistrations that were not covered in the main paper.

Study 2 included an additional exploratory condition (*N* = 273) for a separate line of research as part of a student undergraduate thesis project. As noted in the preregistration, this condition was included to test a separate set of hypotheses and was not intended to be reported with the rest of the package. In essence the extra condition was part of a student project piloting ways to correct information after erroneous information has been shared. This condition does not address the questions in the current paper. For all analyses reported in the manuscript, participants in this exploratory condition were excluded. Participants in this condition were also not included in the numbers we reported for our initial and final samples.

In the interest of full transparency, note that the OSF page for this project also includes a preregistration for a fourth study. While we did collect data for this study, a programming error affected the proper random assignment of several key conditions in the survey (in essence, the programming error resulted in some participants receiving two conditions while others received neither), rendering the collected data invalid. Ultimately, because of budget constraints and because Studies 1-3 already represented a substantial test of the trust models on their own, we opted to report Studies 1-3 in the manuscript while being transparent about the other attempted, but invalid, dataset.

# Manipulation Materials

In all conditions, participants were given a short description of one mainstream source, one counter-mainstream source, and one neutral source. The exact wording of the descriptions was the same across conditions, but varied by study. See Table S1 for a full comparison.

After reading the descriptions of the primary mainstream, counter-mainstream, and neutral sources, participants read a story in which one of those three sources made a serious error in their reporting. Within each study, the topic and the error were the same regardless of which source reported the incorrect information. All studies also included a control condition, in which participants read an irrelevant story about the academy awards, which had nothing to do with health or science topics. The exact wording varied by study and condition. See Table S2 for a full comparison.

## Table S1. Descriptions of primary mainstream, counter-mainstream, and neutral sources preceding manipulation.

| All Studies | | |
| --- | --- | --- |
| In part of this survey you will be asked your thoughts about developing events in the media and about different sources of information.  Below is some information on a few specific information outlets that may be useful while completing this study, so that you know the nature of each information source even if you were not previously familiar with it. Later, you will be asked some questions about some or all of these sources, so please read all of the information carefully. | | |
| Study 1 | Study 2 | Study 3 |
| **Food and Drug Administration (FDA)**  The FDA is a federal public health agency in the United States. It is responsible for the control and supervision of food safety, pharmaceutical drugs (medications), medical devices, and other health-related products. The FDA commissioner reports to the United States Secretary of Health and Human Services.  **National Beacon**  National Beacon is a news site that has often been at odds with mainstream sources. In particular it has drawn controversy for its reporting on health issues, publishing statements from doctors who diverge from scientific consensus.  **Hattrick**  Hattrick is a sports blog that posts daily previews, recaps, and commentaries on major sports stories. It is known for its approachability and conversational tone. In addition to sports stories, it has extended into media and pop culture as well as the occasional article on politics. | **Centers for Disease Control and Prevention (CDC)** The CDC is the national public health agency of the United States. It functions to protect public health and safety through the control and prevention of both infectious and non-infectious disease. The CDC director reports to the United States Secretary of Health and Human Services.   **National Beacon**  National Beacon is a news site that has often been at odds with mainstream sources. In particular it has drawn controversy for its reporting on health issues, publishing statements from doctors who diverge from scientific consensus.  **Hattrick**  Hattrick is a sports blog that posts daily previews, recaps, and commentaries on major sports stories. It is known for its approachability and conversational tone. In addition to sports stories, it has extended into media and pop culture as well as the occasional article on politics. | **The outlets listed below are local to the same state in the USA, but the specific state has been redacted because we want to focus on your general reaction regardless of your opinions about various states.**    **Department of Health**  The Department of Health is the state-level public health agency for **[state name]**. The responsibilities of the agency include oversight of public health services, licensure and training for medical professionals, and public communications on issues of health.  For historical and political reasons, the leadership and senior management of the Department of Health in **[state name]** has been predominantly Democrat-affiliated, and the organization is generally seen as a liberal institution.*    **Today News**  Today News is a news site reporting on stories from **[state name]**. It has often been at odds with mainstream sources, taking a skeptical position towards conventional narratives and institutions. In particular it has drawn controversy for its reporting on health issues, publishing statements from doctors who diverge from scientific consensus.    The reporters and senior management at Today News are predominantly left-leaning and the outlet is generally seen as a liberal organization.*    **Hattrick**  Hattrick is a sports blog that posts daily previews, recaps, and commentaries on major sports stories, with a focus on **[state name]**. It is known for its approachability and conversational tone. In addition to sports stories, it has extended into media and pop culture as well as the occasional article on politics.    The reporters and senior management at Hattrick generally have a left leaning, and the outlet is often seen as liberal.* |

*In Study 3, the political leaning of all three sources was set to be the same as that of the participant, Democrat/liberal or Republican/conservative.

## Table S2. Error manipulation.

|  | Study 1 | Study 2 | Study 3 |
| --- | --- | --- | --- |
| Mainstream Error | In the summer of 2021, following some rumors of health risks, US government officials made a series of statements touting the safety and health benefits of BNH, a chemical compound found in certain packaged foods. The commissioner of the Food and Drug Administration (FDA) tweeted “BNH IS SAFE AND HEALTHY” and stated that “there is NO evidence that the compound has negative effects, and evidence has even shown that it has some important health benefits.” The secretary of health and human services stated that the science did not current support the rumors about BNH and that it was healthy and safe to consume.  The FDA commissioner later deleted his tweet as the Department of Health Human services and federal government removed their guidelines stating the compound was safe.  With current data showing that BNH is linked to numerous negative side effects such as elevated blood pressure and an increased risk of stroke, heart disease, and liver damage, the initial inaccuracy of the FDA and other agencies may have led to poorer quality of health in the United States. | In February and early March of 2020, US government officials instructed people not to wear masks in response to the incoming COVID-19 pandemic. The surgeon general tweeted to “STOP BUYING MASKS” and stated that “They are NOT effective in preventing general public from catching #Coronavirus.” The director of the National Institute of Allergy and Infectious Diseases stated that “there’s no reason to be walking around with a mask.” The Center for Disease Control and Prevention (CDC) did not recommend the use of masks in their guidelines released on March 15.  The surgeon general later deleted his tweet. The CDC and federal government began to recommend that people should wear masks to protect themselves in public.  With current data showing the effectiveness of masks in limiting the transmission of COVID-19, the initial inaccuracy of the CDC and other agencies may have led to a greater spread of the virus. | In the summer of 2021, following some rumors of health risks, officials in the Democrat-led **[state name]** Department of Health made a series of statements touting the safety and health benefits of BNH, a chemical compound found in certain packaged foods. The commissioner of the Department of Health tweeted “BNH IS SAFE AND HEALTHY” and stated that “there is NO evidence that the compound has negative effects, and evidence has even shown that it has some important health benefits.” Another senior official from the agency stated that “there is no reason to avoid food that contains BNH.” In an article posted on August 7th, the **[state name]** Department of Health website stated that the compound was healthy to consume.    The commissioner later deleted their tweet as the **[state name]** Department of Health removed their guidelines stating the compound was safe.    With current data showing that BNH is linked to numerous negative side effects such as elevated blood pressure and an increased risk of stroke, heart disease, and liver damage, the initial inaccuracy of the **[state name]** Department of Health may have led to poorer quality of health in **[state name]** and the United States at large. |
| Counter-Mainstream Error | In the summer of 2021, following some rumors of health risks, news site National Beacon released a series of articles touting the safety and health benefits of BNH, a chemical compound found in certain packaged foods. Headlines told readers that “BNH IS SAFE AND HEALTHY” as articles claimed that “there is NO evidence that the compound has negative effects, and evidence has even shown that it has some important health benefits.” Several editorials were written by “rogue” doctors who stated that the science did not current support the rumors about BNH and that it was healthy and safe to consume. Many of the doctors in question were no longer practicing and some had previously had their licenses revoked.  Later in the year, National Beacon stopped posting about BNH and deleted many of their articles in support of the compound.  With current data showing that BNH is linked to numerous negative side effects such as elevated blood pressure and an increased risk of stroke, heart disease, and liver damage, the inaccuracies spread by National Beacon and similar fringe news sites may have led to poorer quality of health in the United States. | In the summer of 2020, self-described “counter-mainstream” news site National Beacon released a series of articles telling their readers not to wear masks in public during the COVID-19 pandemic. Headlines implored readers to “STOP WEARING MASKS” and claimed that “They are NOT effective in any way.” Several editorials were written by “rogue” doctors who claimed that the scientific consensus was wrong and that wearing a mask was a meaningless gesture. Articles criticized the mainstream perception that masks were effective.  National Beacon later deleted many of their previous anti-mask articles. They released a statement saying that although they were “deeply committed to criticizing mainstream institutions and narratives”, people should wear masks to protect themselves in public.  With current data showing the effectiveness of masks in limiting the transmission of COVID-19, the inaccuracies spread by National Beacon and similar fringe news sites may have led to a greater spread of the virus | In the summer of 2021, following some rumors of health risks, liberal news site Today News released a series of articles touting the safety and health benefits of BNH, a chemical compound found in certain packaged foods. Headlines told readers that “BNH IS SAFE AND HEALTHY” as articles claimed that “there is NO evidence that the compound has negative effects, and evidence has even shown that it has some important health benefits.” Several editorials were written by “rogue” doctors who claimed that the scientific consensus was wrong and that BNH was healthy and safe to consume. Many of the doctors in question were no longer practicing and some had previously had their licenses revoked.  Later in the year, Today News stopped posting about BNH and deleted many of their articles in support of the compound.  With current data showing that BNH is linked to numerous negative side effects such as elevated blood pressure and an increased risk of stroke, heart disease, and liver damage, the inaccuracies spread by Today News and similar fringe news sites may have led to poorer quality of health in **[state name]** and the United States at large. |
| Neutral Error | In the summer of 2021, following some rumors of health risks, sports blog Hattrick released several articles touting the safety and health benefits of BNH, a chemical compound found in certain packaged foods. Headlines told readers that “BNH IS SAFE AND HEALTHY” as articles claimed that “there is NO evidence that the compound has negative effects, and evidence has even shown that it has some important health benefits.” Several reports written by the site’s sports reporters stated that the science did not currently support the rumors about BNH and that it was healthy and safe to consume.   Later in the year, Hattrick stopped posting about BNH and deleted many of their articles in support of the compound.  With current data showing that BNH is linked to numerous negative side effects such as elevated blood pressure and an increased risk of stroke, heart disease, and liver damage, the inaccuracies spread by Hattrick and other sources may have led to poorer quality of health in the United States. | In the summer of 2020, sports blog Hattrick released several articles telling their readers they did not need to wear masks in public during the COVID-19 pandemic. Headlines told readers to “STOP WEARING MASKS” and claimed that “They are not effective in protecting people.” Several reports written by the site’s sports reporters stated that the science did not currently support the necessity of mask use at major events. The editorial staff did not recommend the use of masks in their guidelines released on the site.  Hattrick later deleted many of their previous anti-mask articles. They released a statement saying that people should wear masks to protect themselves in public.  With current data showing the effectiveness of masks in limiting the transmission of COVID-19, the inaccuracies spread by Hattrick and other sources and may have led to a greater spread of the virus. | Condition Not Included |
| Control | The 94th Academy Awards were held on March 27, 2022. While feel-good film CODA took the spotlight with awards for best picture and best adapted screenplay, the movie to rack up the most trophies was sci-fi epic Dune. Dune took six awards in total, including best original score, best sound, best production design, best cinematography, best film editing, and best visual effects.  The success of Dune was not guaranteed when director Dennis Villeneuve began production in 2019. Part one of a two-part series, the second installment was not confirmed until the $165 million project grossed over $400 million worldwide. Part two is set to release in October 2023 and cover the remaining story from the book on which it is based. | The 94th Academy Awards were held on March 27, 2022. While feel-good film CODA took the spotlight with awards for best picture and best adapted screenplay, the movie to rack up the most trophies was sci-fi epic Dune. Dune took six awards in total, including best original score, best sound, best production design, best cinematography, best film editing, and best visual effects.  The success of Dune was not guaranteed when director Dennis Villeneuve began production in 2019. Part one of a two-part series, the second installment was not confirmed until the $165 million project grossed over $400 million worldwide. Part two is set to release in October 2023 and cover the remaining story from the book on which it is based. | The 94th Academy Awards were held on March 27, 2022. While feel-good film CODA took the spotlight with awards for best picture and best adapted screenplay, the movie to rack up the most trophies was sci-fi epic Dune. Dune took six awards in total, including best original score, best sound, best production design, best cinematography, best film editing, and best visual effects.  The success of Dune was not guaranteed when director Dennis Villeneuve began production in 2019. Part one of a two-part series, the second installment was not confirmed until the $165 million project grossed over $400 million worldwide. Part two is set to release in October 2023 and cover the remaining story from the book on which it is based. |

In Study 3, the political leaning of all three sources was set to be the same as that of the participant, Democrat/liberal or Republican/conservative.

# Excluding Participants Based on Memory Checks

In the main analyses reported in the manuscript, we report the percentage of participants who correctly answered each of the memory checks, but we do not exclude participants from the sample based on these checks. Here we do just that, examining whether our results remain the same when we only include participants who correctly answered *all* of the checks. In all three studies, participants were excluded based on the four checks, including which source made the error, the source’s initial (erroneous) stance on the issue at hand, whether the source retracted or reversed their stance, and what the current data indicates about the issue The original exclusion criteria reported in the main text was still applied as well. Because our inclusion criteria were so stringent (answering correctly on every single memory check), samples were substantially smaller in each study, *N* = 526, 668, and 802, respectively.

Results of this analysis are reported in Tables S3-5. Overall, the results are not substantially different from the original analyses. The main point of difference is that in contrast to the main analyses, we did not find any three-way interactions in Studies 2 or 3, possibly due to the reduced sample size.

## Table S3. Mixed ANOVA for primary trust measures with only participants who correctly answered all memory checks, estimated marginal means and standard errors, Study 1.

| **Source** | | | | | | | | | | | | | | | | | |
| --- | --- | --- | --- | --- | --- | --- | --- | --- | --- | --- | --- | --- | --- | --- | --- | --- | --- |
| Main Effect | | | | | | Mainstream | | | | Counter-Mainstream | | | | | Neutral | | |
| *F*(2, 1036) = 505.44, η_p_^2^ = .49*** | | | | | | 5.01_a_  (0.06) | | | | 2.62_b_  (0.06) | | | | | 3.15_c_  (0.06) | | |
| **Error Condition** | | | | | | | | | | | | | | | | | |
| Main Effect | | | | Mainstream Error | | | | | Counter-Mainstream Error | | | Neutral Error | | | | | Control |
| *F*(3, 518) = 29.18, η_p_^2^ = .15*** | | | | 3.58_a_  (0.09) | | | | | 3.53_a_  (0.08) | | | 3.17_b_  (0.08) | | | | | 4.10_c_  (0.06) |
| **Political Party** | | | | | | | | | | | | | | | | | |
| Main Effect | | | | | Republican | | | | | | | | Democrat | | | | |
| *F*(1, 518) = 5.20, η_p_^2^ = .01* | | | | | 3.50_a_  (0.06) | | | | | | | | 3.68_b_  (0.05) | | | | |
| **Source × Error Condition** | | | | | | | | | | | | | | | | | |
| 2-Way Interaction |  | | Mainstream Error | | | | | Counter-Mainstream Error | | | Neutral Error | | | | | Control | |
| *F*(6, 1036) = 30.41, η_p_^2^ = .15*** | Mainstream | | 4.53_a_  (0.13) | | | | | 5.22_b_  (0.13) | | | 5.19_b_  (0.13) | | | | | 5.10_b_  (0.10) | |
|  | Counter-Mainstream | | 2.81_a_  (0.13) | | | | | 1.96_b_  (0.13) | | | 2.46_a_  (0.13) | | | | | 3.24_c_  (0.10) | |
|  | Neutral | | 3.39_a_  (0.13) | | | | | 3.42_a_  (0.12) | | | 1.85_b_  (0.12) | | | | | 3.95_c_  (0.09) | |
| **Source × Political Party** | | | | | | | | | | | | | | | | | |
| 2- Way Interaction | |  | | | | | Republican | | | | | | | Democrat | | | |
| *F*(6, 1036) = 93.33, η_p_^2^ = .15*** | | Mainstream | | | | | 4.31_a_  (0.09) | | | | | | | 5.71_b_  (0.08) | | | |
|  |  | Counter-Mainstream | | | | | 2.95_a_  (0.09) | | | | | | | 2.29_b_  (0.08) | | | |
|  |  | Neutral | | | | | 3.25_a_  (0.09) | | | | | | | 3.05_a_  (0.08) | | | |
| **Error Condition × Political Party** | | | | | | | | | | | | | | | | | |
| 2-Way Interaction |  | | Mainstream Error | | | | | Counter-Mainstream Error | | | Neutral Error | | | | | Control | |
| *F*(3, 518) = 1.54,  η_p_^2^ = .01 | Republican | | 3.64_ab_  (0.13) | | | | | 3.42_a_  (0.12) | | | 3.01_c_  (0.13) | | | | | 3.95_b_  (0.09) | |
|  | Democrat | | 3.52_ab_  (0.12) | | | | | 3.65_a_  (0.12) | | | 3.32_b_  (0.10) | | | | | 4.25_c_  (0.08) | |
| **Source × Error Condition × Political Party** | | | | | | | | | | | | | | | | | |
| 3-Way Interaction | | | | | | | | | | | | | | | | | |
| *F*(6, 1036) = 15.1, η_p_^2^ = .01 | | | | | | | | | | | | | | | | | |

Within each row, means for each condition that do not share a subscript are significantly different. Subscripts do not compare between rows. †p < .10, * p < .05, ** p < .01, *** p < .001

## Table S4. Mixed ANOVA for primary trust measures with only participants who correctly answered all memory checks, estimated marginal means and standard errors, Study 2.

| **Source** | | | | | | | | | | | | | | | | | |
| --- | --- | --- | --- | --- | --- | --- | --- | --- | --- | --- | --- | --- | --- | --- | --- | --- | --- |
| Main Effect | | | | | | Mainstream | | | | Counter-Mainstream | | | | | Neutral | | |
| *F*(2, 1320) = 712.87, η_p_^2^ = .52*** | | | | | | 5.02_a_  (0.06) | | | | 2.69_b_  (0.06) | | | | | 2.73_b_  (0.05) | | |
| **Error Condition** | | | | | | | | | | | | | | | | | |
| Main Effect | | | | Mainstream Error | | | | | Counter-Mainstream Error | | | Neutral Error | | | | | Control |
| *F*(3, 660) = 15.29, η_p_^2^ = .07*** | | | | 3.44_a_  (0.07) | | | | | 3.35_a_  (0.07) | | | 3.31_a_  (0.07) | | | | | 3.83_b_  (0.06) |
| **Political Party** | | | | | | | | | | | | | | | | | |
| Main Effect | | | | | Republican | | | | | | | | Democrat | | | | |
| *F*(1, 660) = 8.67, η_p_^2^ = .01** | | | | | 3.38_a_  (0.05) | | | | | | | | 3.58_b_  (0.04) | | | | |
| **Source × Error Condition** | | | | | | | | | | | | | | | | | |
| 2-Way Interaction |  | | Mainstream Error | | | | | Counter-Mainstream Error | | | Neutral Error | | | | | Control | |
| *F*(6, 1320) = 16.76, η_p_^2^ = .07*** | Mainstream | | 4.66_a_  (0.12) | | | | | 5.21_b_  (0.12) | | | 5.12_b_  (0.12) | | | | | 5.08_b_  (0.09) | |
|  | Counter-Mainstream | | 2.92_a_  (0.12) | | | | | 2.03_b_  (0.12) | | | 2.70_a_  (0.12) | | | | | 3.22_c_  (0.09) | |
|  | Neutral | | 2.85_a_  (0.10) | | | | | 2.80_a_  (0.10) | | | 2.10_b_  (0.10) | | | | | 3.18_c_  (0.08) | |
| **Source × Political Party** | | | | | | | | | | | | | | | | | |
| 2- Way Interaction | |  | | | | | Republican | | | | | | | Democrat | | | |
| *F*(2, 1320) = 173.61, η_p_^2^ = .21*** | | Mainstream | | | | | 4.18_a_  (0.09) | | | | | | | 5.85_b_  (0.07) | | | |
|  |  | Counter-Mainstream | | | | | 3.13_a_  (0.09) | | | | | | | 2.26_b_  (0.07) | | | |
|  |  | Neutral | | | | | 2.83_a_  (0.07) | | | | | | | 2.64_b_  (0.06) | | | |
| **Error Condition × Political Party** | | | | | | | | | | | | | | | | | |
| 2-Way Interaction |  | | Mainstream Error | | | | | Counter-Mainstream Error | | | Neutral Error | | | | | Control | |
| *F*(3, 660) = 2.50, η_p_^2^ = .01† | Republican | | 3.36_ab_  (0.11) | | | | | 3.24_a_  (0.12) | | | 3.32_a_  (0.11) | | | | | 3.60_b_  (0.09) | |
|  | Democrat | | 3.52_a_  (0.10) | | | | | 3.45_a_  (0.09) | | | 3.29_a_  (0.09) | | | | | 4.06_b_  (0.08) | |
| **Source × Error Condition × Political Party** | | | | | | | | | | | | | | | | | |
| 3-Way Interaction | | | | | | | | | | | | | | | | | |
| *F*(6, 1320) = 1.54, η_p_^2^ = .01 | | | | | | | | | | | | | | | | | |

Within each row, means for each condition that do not share a subscript are significantly different. Subscripts do not compare between rows. †p < .10, * p < .05, ** p < .01, *** p < .001

## Table S5. Mixed ANOVA for primary trust measures with only participants who correctly answered all memory checks, estimated marginal means and standard errors, Study 3.

| **Source** | | | | | | | | | | | | | | | | | |
| --- | --- | --- | --- | --- | --- | --- | --- | --- | --- | --- | --- | --- | --- | --- | --- | --- | --- |
| Main Effect | | | | | | Mainstream | | | | Counter-Mainstream | | | | | Neutral | | |
| *F*(2, 1592) = 156.40, η_p_^2^ = .16*** | | | | | | 4.55_a_  (0.05) | | | | 3.54_b_  (0.05) | | | | | 4.08_c_  (0.05) | | |
| **Error Condition** | | | | | | | | | | | | | | | | | |
| Main Effect | | | | Mainstream Error | | | | | Counter-Mainstream Error | | | Neutral Error | | | | | Control |
| *F*(2, 796) = 112.45, η_p_^2^ = .22*** | | | | 3.77_a_  (0.07) | | | | | 3.67_a_  (0.07) | | | — | | | | | 4.72_b_  (0.05) |
| **Political Party** | | | | | | | | | | | | | | | | | |
| Main Effect | | | | | Republican | | | | | | | | Democrat | | | | |
| *F*(1, 796) = 8.32, η_p_^2^ = .01** | | | | | 3.96_a_  (0.05) | | | | | | | | 4.16_b_  (0.05) | | | | |
| **Source × Error Condition** | | | | | | | | | | | | | | | | | |
| 2-Way Interaction |  | | Mainstream Error | | | | | Counter-Mainstream Error | | | Neutral Error | | | | | Control | |
| *F*(4, 1592) = 84.55, η_p_^2^ = .18*** | Mainstream | | 3.57_a_  (0.09) | | | | | 4.88_b_  (0.09) | | | — | | | | | 5.22_c_  (0.07) | |
|  | Counter-Mainstream | | 3.81_a_  (0.09) | | | | | 2.42_b_  (0.09) | | | — | | | | | 4.40_c_  (0.07) | |
|  | Neutral | | 3.92_a_  (0.09) | | | | | 3.77_a_  (0.09) | | | — | | | | | 4.55_b_  (0.06) | |
| **Source × Political Party** | | | | | | | | | | | | | | | | | |
| 2- Way Interaction | |  | | | | | Republican | | | | | | | Democrat | | | |
| *F*(2, 1592) = 50.62,  η_p_^2^ = .06*** | | Mainstream | | | | | 4.12_a_  (0.07) | | | | | | | 4.99_b_  (0.07) | | | |
|  |  | Counter-Mainstream | | | | | 3.63_a_  (0.07) | | | | | | | 3.46_a_  (0.07) | | | |
|  |  | Neutral | | | | | 4.13_a_  (0.07) | | | | | | | 4.03_a_  (0.07) | | | |
| **Error Condition × Political Party** | | | | | | | | | | | | | | | | | |
| 2-Way Interaction |  | | Mainstream Error | | | | | Counter-Mainstream Error | | | Neutral Error | | | | | Control | |
| *F*(2, 796) = 0.96,  η_p_^2^ = .002 | Republican | | 3.68_a_  (0.09) | | | | | 3.64_a_  (0.10) | | | — | | | | | 4.56_b_  (0.07) | |
|  | Democrat | | 3.86_a_  (0.09) | | | | | 3.74_a_  (0.09) | | | — | | | | | 4.88_b_  (0.07) | |
| **Source × Error Condition × Political Party** | | | | | | | | | | | | | | | | | |
| 3-Way Interaction | | | | | | | | | | | | | | | | | |
| *F*(4, 1592) = 2,14, η_p_^2^ = .01 | | | | | | | | | | | | | | | | | |

Within each row, means for each condition that do not share a subscript are significantly different. Subscripts do not compare between rows. †p < .10, * p < .05, ** p < .01, *** p < .001

# Main Analyses — Univariate ANOVAs

In our original preregistered analyses for all three studies, we had intended to run a separate univariate between-subjects ANOVA for each of our three primary trust measures (mainstream, counter-mainstream, and neutral). However, to account for additional within-subject variance, we decided to use a mixed model instead for our main analyses. Here we report the results of the originally planned between-subjects univariate ANOVAs in Tables S6 & S7. Overall, the main pattern of results is not substantially different from that of the mixed ANOVAs.

## Table S6. Separate univariate ANOVAs for primary trust measures, estimated marginal means and standard errors.

| Study 1 | | | | | | | | | | |
| --- | --- | --- | --- | --- | --- | --- | --- | --- | --- | --- |
|  | Main Effect  Political Party | Republican | Democrat | Main Effect Manipulation | Mainstream Error | Counter-Mainstream Error | Neutral Error | Control | Political Party × Manipulation |  |
| Mainstream Trust | *F*(1, 779) = 131.08,  η_p_^2^ = .14*** | 4.43  (0.07) | 5.58  (0.07) | *F*(3, 779) = 3.74,  η_p_^2^ = .01* | 4.71_a_  (0.10) | 5.07_b_  (0.10) | 5.13_b_  (0.10) | 5.10_b_  (0.10) | *F*(3, 779) = 0.50,  η_p_^2^ = .002 |  |
| Counter-Mainstream Trust | *F*(1, 779) = 38.21,  η_p_^2^ = .05*** | 3.24  (0.08) | 2.60  (0.07) | *F*(3, 779) = 12.55,  η_p_^2^ = .05*** | 3.15_ac_  (0.10) | 2.42_b_  (0.10) | 2.88_c_  (0.11) | 3.24_a_  (0.11) | *F*(3, 779) = 0.01,  η_p_^2^ < .001 |  |
| Neutral Trust | *F*(1, 779) = 4.29,  η_p_^2^ = .01* | 3.41  (0.07) | 3.21  (0.07) | *F*(3, 779) = 47.56,  η_p_^2^ = .16*** | 3.45_a_  (0.10) | 3.48_a_  (0.10) | 2.36_b_  (0.10) | 3.95_c_  (0.10) | *F*(3, 779) = 2.27,  η_p_^2^ = .01† |  |
| Study 2 | | | | | | | | | | |
|  | Main Effect  Political Party | Republican | Democrat | Main Effect Manipulation | Mainstream Error | Counter-Mainstream Error | Neutral Error | Control | Political Party × Manipulation |  |
| Mainstream Trust | *F*(1, 934) = 367.76,  η_p_^2^ = .28*** | 4.00  (0.07) | 5.83  (0.06) | *F*(3, 934) = 2.72,  η_p_^2^ = .01* | 4.74_a_  (0.10) | 5.00_b_  (0.09) | 4.83_ab_  (0.10) | 5.08_b_  (0.10) | *F*(3, 934) = 2.10,  η_p_^2^ = .01† |  |
| Counter-Mainstream Trust | *F*(1, 934) = 50.11,  η_p_^2^ = .05*** | 3.31  (0.08) | 2.59  (0.07) | *F*(3, 934) = 12.22,  η_p_^2^ = .04*** | 3.11_a_  (0.10) | 2.43_b_  (0.10) | 3.04_a_  (0.10) | 3.22_a_  (0.10) | *F*(3, 934) = 2.41,  η_p_^2^ = .01† |  |
| Neutral Trust | *F*(1, 934) = 1.14,  η_p_^2^ = .001 | 2.99  (0.07) | 2.89  (0.06) | *F*(3, 934) = 12.81,  η_p_^2^ = .04*** | 3.14_a_  (0.09) | 2.95_a_  (0.09) | 2.48_b_  (0.09) | 3.18_a_  (0.09) | *F*(3, 934) = 2.30,  η_p_^2^ = .01† |  |
| Study 3 | | | | | | | | | | |
|  | Main Effect  Political Party | Republican | Democrat | Main Effect Manipulation | Mainstream Error | Counter-Mainstream Error | Neutral Error | Control | Political Party × Manipulation |  |
| Mainstream Trust | *F*(1, 1216) = 95.66,  η_p_^2^ = .07*** | 4.20  (0.06) | 4.99  (0.06) | *F*(2, 1216) = 90.11,  η_p_^2^ = .13*** | 3.90_a_  (0.07) | 4.66_b_  (0.07) | — | 5.22_c_  (0.07) | *F*(2, 1216) = 2.41,  η_p_^2^ = .004† |  |
| Counter-Mainstream Trust | *F*(1, 1216) = .04,  η_p_^2^ < .001† | 3.79  (0.06) | 3.78  (0.06) | *F*(2, 1216) = 115.92,  η_p_^2^ = .16*** | 4.00_a_  (0.07) | 2.95_b_  (0.07) | — | 4.40_c_  (0.07) | *F*(2, 1216) = 4.47,  η_p_^2^ = .01* |  |
| Neutral Trust | *F*(3, 1216) = 0.59,  η_p_^2^ < .001 | 4.06  (0.05) | 4.12  (0.05) | *F*(2, 1216) = 39.66,  η_p_^2^ = .06*** | 3.96_a_  (0.07) | 3.75_b_  (0.07) | — | 4.55_c_  (0.07) | *F*(2, 1216) = 2.14,  η_p_^2^ = .004 |  |

Within each row, means for each condition that do not share a subscript are significantly different. Subscripts do not compare between rows. †p < .10, * p < .05, ** p < .01, *** p < .001

## Table S7. Simple main effects for significant condition × political party interaction on counter-mainstream trust, estimated marginal means and standard errors, Study 3.

| Republican | | |
| --- | --- | --- |
| Mainstream Error | Counter-Mainstream Error | Control |
| 3.86_a_  (0.10) | 3.11_b_  (0.10) | 4.40_c_  (0.10) |
| Democrat | | |
| Mainstream Error | Counter-Mainstream Error | Control |
| 4.14_a_  (0.10) | 2.80_b_  (0.10) | 4.40_a_  (0.10) |

Within each row, means for each condition that do not share a subscript are significantly different. Subscripts do not compare between rows. †p < .10, * p < .05, ** p < .01, *** p < .001

# Likelihood of Using Source (Single Item Analyses)

When someone loses trust in a source, they may not begin to *trust* other sources more, as only the originally source has done something to prompt a change in their perceived reliability. However, because the original source may not be able to be relied upon (at least to the same extent), people may feel inclined to *use* other sources more, even if their level of trust in them stays the same. For this reason, we considered whether our results would be the same if we only examined the final item in each of our trust measures: “How likely are you to use [source] as a source of information in general?” The same mixed ANOVA was employed for each study, only we only used the single use-based item for each source. The results, presented in Tables S8-10, do not substantially different from those in the main analyses.

## Table S8. Mixed ANOVA for likelihood of using source, estimated marginal means and standard errors, Study 1.

| **Source** | | | | | | | | | | | | | | | | | |
| --- | --- | --- | --- | --- | --- | --- | --- | --- | --- | --- | --- | --- | --- | --- | --- | --- | --- |
| Main Effect | | | | | | Mainstream | | | | Counter-Mainstream | | | | | Neutral | | |
| *F*(2, 1558) = 632.92, η_p_^2^ = .45*** | | | | | | 5.00_a_  (0.06) | | | | 2.68_b_  (0.06) | | | | | 2.90_c_  (0.06) | | |
| **Error Condition** | | | | | | | | | | | | | | | | | |
| Main Effect | | | | Mainstream Error | | | | | Counter-Mainstream Error | | | Neutral Error | | | | | Control |
| *F*(3, 779) = 7.26, η_p_^2^ = .03*** | | | | 3.52_a_  (0.08) | | | | | 3.45_a_  (0.08) | | | 3.33_a_  (0.08) | | | | | 3.82_b_  (0.08) |
| **Political Party** | | | | | | | | | | | | | | | | | |
| Main Effect | | | | | Republican | | | | | | | | Democrat | | | | |
| *F*(1, 779) = 2.58, η_p_^2^ = .003 | | | | | 3.46_a_  (0.06) | | | | | | | | 3.59_a_  (0.05) | | | | |
| **Source × Error Condition** | | | | | | | | | | | | | | | | | |
| 2-Way Interaction |  | | Mainstream Error | | | | | Counter-Mainstream Error | | | Neutral Error | | | | | Control | |
| *F*(6, 1558) = 17.13, η_p_^2^ = .06*** | Mainstream | | 4.68_a_  (0.11) | | | | | 5.07_b_  (0.11) | | | 5.19_b_  (0.11) | | | | | 5.05_b_  (0.11) | |
|  | Counter-Mainstream | | 2.92_a_  (0.12) | | | | | 2.21_b_  (0.12) | | | 2.67_a_  (0.12) | | | | | 2.94_a_  (0.12) | |
|  | Neutral | | 2.95_a_  (0.11) | | | | | 3.06_a_  (0.11) | | | 2.11_b_  (0.12) | | | | | 3.48_c_  (0.12) | |
| **Source × Political Party** | | | | | | | | | | | | | | | | | |
| 2- Way Interaction | |  | | | | | Republican | | | | | | | Democrat | | | |
| *F*(2, 1558) = 94.46,  η_p_^2^ = .11*** | | Mainstream | | | | | 4.37_a_  (0.08) | | | | | | | 5.63_b_  (0.08) | | | |
|  |  | Counter-Mainstream | | | | | 2.98_a_  (0.08) | | | | | | | 2.38_b_  (0.08) | | | |
|  |  | Neutral | | | | | 3.04_a_  (0.08) | | | | | | | 2.76_b_  (0.08) | | | |
| **Error Condition × Political Party** | | | | | | | | | | | | | | | | | |
| 2-Way Interaction |  | | Mainstream Error | | | | | Counter-Mainstream Error | | | Neutral Error | | | | | Control | |
| *F*(3, 779) = 1.20,  η_p_^2^ = .01 | Republican | | 3.52_a_  (0.11) | | | | | 3.37_a_  (0.11) | | | 3.33_a_  (0.12) | | | | | 3.64_a_  (0.12) | |
|  | Democrat | | 3.52_a_  (0.11) | | | | | 3.52_a_  (0.11) | | | 3.32_a_  (0.11) | | | | | 4.00_b_  (0.11) | |
| **Source × Error Condition × Political Party** | | | | | | | | | | | | | | | | | |
| 3-Way Interaction | | | | | | | | | | | | | | | | | |
| *F*(6, 1558) = 0.78, η_p_^2^ = .003 | | | | | | | | | | | | | | | | | |

Within each row, means for each condition that do not share a subscript are significantly different. Subscripts do not compare between rows. †p < .10, * p < .05, ** p < .01, *** p < .001

## Table S9. Mixed ANOVA for likelihood of using source, estimated marginal means and standard errors, Study 2.

| **Source** | | | | | | | | | | | | | | | | | |
| --- | --- | --- | --- | --- | --- | --- | --- | --- | --- | --- | --- | --- | --- | --- | --- | --- | --- |
| Main Effect | | | | | | Mainstream | | | | Counter-Mainstream | | | | | Neutral | | |
| *F*(2, 1868) = 798.77, η_p_^2^ = .46*** | | | | | | 4.95_a_  (0.05) | | | | 2.76_b_  (0.05) | | | | | 2.61_c_  (0.05) | | |
| **Error Condition** | | | | | | | | | | | | | | | | | |
| Main Effect | | | | Mainstream Error | | | | | Counter-Mainstream Error | | | Neutral Error | | | | | Control |
| *F*(3, 934) = 4.21, η_p_^2^ = .01** | | | | 3.46_ab_  (0.07) | | | | | 3.32_a_  (0.07) | | | 3.34_a_  (0.07) | | | | | 3.63_b_  (0.07) |
| **Political Party** | | | | | | | | | | | | | | | | | |
| Main Effect | | | | | Republican | | | | | | | | Democrat | | | | |
| *F*(1, 934) = 23.72, η_p_^2^ = .03*** | | | | | 3.27_a_  (0.05) | | | | | | | | 3.61_b_  (0.05) | | | | |
| **Source × Error Condition** | | | | | | | | | | | | | | | | | |
| 2-Way Interaction |  | | Mainstream Error | | | | | Counter-Mainstream Error | | | Neutral Error | | | | | Control | |
| *F*(6, 1868) = 7.32, η_p_^2^ = .02*** | Mainstream | | 4.75_a_  (0.10) | | | | | 5.07_b_  (0.10) | | | 4.86_ab_  (0.10) | | | | | 5.14_b_  (0.10) | |
|  | Counter-Mainstream | | 2.89_a_  (0.11) | | | | | 2.29_b_  (0.11) | | | 2.88_a_  (0.11) | | | | | 2.98_a_  (0.11) | |
|  | Neutral | | 2.75_a_  (0.10) | | | | | 2.61_a_  (0.10) | | | 2.28_b_  (0.10) | | | | | 2.79_a_  (0.10) | |
| **Source × Political Party** | | | | | | | | | | | | | | | | | |
| 2- Way Interaction | |  | | | | | Republican | | | | | | | Democrat | | | |
| *F*(2, 1868) = 228.28, η_p_^2^ = .20*** | | Mainstream | | | | | 3.99_a_  (0.08) | | | | | | | 5.92_b_  (0.07) | | | |
|  |  | Counter-Mainstream | | | | | 3.12_a_  (0.08) | | | | | | | 2.40_b_  (0.07) | | | |
|  |  | Neutral | | | | | 2.70_a_  (0.07) | | | | | | | 2.51_a_  (0.07) | | | |
| **Error Condition × Political Party** | | | | | | | | | | | | | | | | | |
| 2-Way Interaction |  | | Mainstream Error | | | | | Counter-Mainstream Error | | | Neutral Error | | | | | Control | |
| *F*(3, 934) = 0.41,  η_p_^2^ = .001 | Republican | | 3.33_a_  (0.10) | | | | | 3.19_a_  (0.10) | | | 3.16_a_  (0.10) | | | | | 3.41_a_  (0.11) | |
|  | Democrat | | 3.59_a_  (0.09) | | | | | 3.46_a_  (0.09) | | | 3.53_a_  (0.10) | | | | | 3.86_b_  (0.09) | |
| **Source × Error Condition × Political Party** | | | | | | | | | | | | | | | | | |
| 3-Way Interaction | | | | | | | | | | | | | | | | | |
| *F*(6, 1868) = 3.01, η_p_^2^ = .01* | | | | | | | | | | | | | | | | | |

| **Mainstream Source** | | | | | |
| --- | --- | --- | --- | --- | --- |
| Simple  2-Way Interaction |  | Mainstream Error | Counter-Mainstream Error | Neutral Error | Control |
| *F*(3, 934) = 2.40,  η_p_^2^ = .01† | Republican | 3.98_ab_  (0.15) | 4.00_ab_  (0.15) | 3.74_a_  (0.15) | 4.23_b_  (0.16) |
|  | Democrat | 5.52_a_  (0.14) | 6.13_b_  (0.14) | 5.98_b_  (0.14) | 6.04_b_  (0.14) |
| **Counter-Mainstream Source** | | | | | |
| Simple  2-Way Interaction |  | Mainstream Error | Counter-Mainstream Error | Neutral Error | Control |
| *F*(3, 934) = 1.94,  η_p_^2^ = .01 | Republican | 3.23_a_  (0.16) | 2.86_a_  (0.16) | 3.21_a_  (0.16) | 3.19_a_  (0.16) |
|  | Democrat | 2.54_a_  (0.14) | 1.72_b_  (0.15) | 3.21_a_  (0.16) | 3.19_a_  (0.16) |
| **Neutral Source** | | | | | |
| Simple  2-Way Interaction |  | Mainstream Error | Counter-Mainstream Error | Neutral Error | Control |
| *F*(3, 934) = 1.04,  η_p_^2^ = .003 | Republican | 2.78_a_  (0.15) | 2.70_a_  (0.14) | 2.52_a_  (0.13) | 2.81_a_  (0.15) |
|  | Democrat | 2.72_a_  (0.13) | 2.52_a_  (0.13) | 2.05_b_  (0.14) | 2.77_a_  (0.13) |

Within each row, means for each condition that do not share a subscript are significantly different. Subscripts do not compare between rows. †p < .10, * p < .05, ** p < .01, *** p < .001

## Table S10. Mixed ANOVA for likelihood of using source, estimated marginal means and standard errors, Study 3.

| **Source** | | | | | | | | | | | | | | | | | |
| --- | --- | --- | --- | --- | --- | --- | --- | --- | --- | --- | --- | --- | --- | --- | --- | --- | --- |
| Main Effect | | | | | | Mainstream | | | | Counter-Mainstream | | | | | Neutral | | |
| *F*(2, 2424) = 164.03, η_p_^2^ = .12*** | | | | | | 4.59_a_  (0.04) | | | | 3.69_b_  (0.05) | | | | | 3.85_c_  (0.05) | | |
| **Error Condition** | | | | | | | | | | | | | | | | | |
| Main Effect | | | | Mainstream Error | | | | | Counter-Mainstream Error | | | Neutral Error | | | | | Control |
| *F*(2, 1212) = 75.27, η_p_^2^ = .11*** | | | | 3.85_a_  (0.06) | | | | | 3.68_a_  (0.06) | | | — | | | | | 4.60_b_  (0.06) |
| **Political Party** | | | | | | | | | | | | | | | | | |
| Main Effect | | | | | Republican | | | | | | | | Democrat | | | | |
| *F*(1, 1212) = 15.06, η_p_^2^ = .01*** | | | | | 3.92_a_  (0.05) | | | | | | | | 4.17_b_  (0.05) | | | | |
| **Source × Error Condition** | | | | | | | | | | | | | | | | | |
| 2-Way Interaction |  | | Mainstream Error | | | | | Counter-Mainstream Error | | | Neutral Error | | | | | Control | |
| *F*(4, 2424) = 51.87, η_p_^2^ = .08*** | Mainstream | | 3.90_a_  (0.08) | | | | | 4.65_b_  (0.08) | | | — | | | | | 5.22_c_  (0.08) | |
|  | Counter-Mainstream | | 3.92_a_  (0.08) | | | | | 2.85_b_  (0.08) | | | — | | | | | 4.31_c_  (0.08) | |
|  | Neutral | | 3.72_a_  (0.08) | | | | | 3.55_a_  (0.08) | | | — | | | | | 4.29_b_  (0.08) | |
| **Source × Political Party** | | | | | | | | | | | | | | | | | |
| 2- Way Interaction | |  | | | | | Republican | | | | | | | Democrat | | | |
| *F*(2, 2424) = 46.54,  η_p_^2^ = .04*** | | Mainstream | | | | | 4.17_a_  (0.06) | | | | | | | 5.01_b_  (0.06) | | | |
|  |  | Counter-Mainstream | | | | | 3.72_a_  (0.07) | | | | | | | 3.66_b_  (0.06) | | | |
|  |  | Neutral | | | | | 3.86_a_  (0.07) | | | | | | | 3.84_b_  (0.06) | | | |
| **Error Condition × Political Party** | | | | | | | | | | | | | | | | | |
| 2-Way Interaction |  | | Mainstream Error | | | | | Counter-Mainstream Error | | | Neutral Error | | | | | Control | |
| *F*(2, 1212) = 1.15, η_p_^2^ = .002 | Republican | | 3.68_a_  (0.08) | | | | | 3.63_a_  (0.08) | | | — | | | | | 4.44_b_  (0.08) | |
|  | Democrat | | 4.01_a_  (0.08) | | | | | 3.74_b_  (0.08) | | | — | | | | | 4.77_c_  (0.08) | |
| **Source × Error Condition × Political Party** | | | | | | | | | | | | | | | | | |
| 3-Way Interaction | | | | | | | | | | | | | | | | | |
| *F*(4, 2424) = 5.19, η_p_^2^ = .01*** | | | | | | | | | | | | | | | | | |

| **Mainstream Source** | | | | | |
| --- | --- | --- | --- | --- | --- |
| Simple  2-Way Interaction |  | Mainstream Error | Counter-Mainstream Error | Neutral Error | Control |
| *F*(2, 1215) = 3.53,  η_p_^2^ = .01* | Republican | 3.62_a_  (0.11) | 4.21_b_  (0.11) | — | 4.67_c_  (0.11) |
|  | Democrat | 4.16_a_  (0.11) | 5.11_b_  (0.11) | — | 5.77_c_  (0.11) |
| **Counter-Mainstream Source** | | | | | |
| Simple  2-Way Interaction |  | Mainstream Error | Counter-Mainstream Error | Neutral Error | Control |
| *F*(2, 1214) = 3.14,  η_p_^2^ = .01* | Republican | 3.81_a_  (0.11) | 3.02_b_  (0.11) | — | 4.34_c_  (0.11) |
|  | Democrat | 4.03_a_  (0.11) | 2.69_b_  (0.11) | — | 4.27_a_  (0.11) |
| **Neutral Source** | | | | | |
| Simple  2-Way Interaction |  | Mainstream Error | Counter-Mainstream Error | Neutral Error | Control |
| *F*(2, 1215) = 2.09,  η_p_^2^ = .003 | Republican | 3.59_a_  (0.11) | 3.65_a_  (0.11) | — | 4.34_b_  (0.11) |
|  | Democrat | 3.83_a_  (0.11) | 3.45_b_  (0.11) | — | 4.25_c_  (0.11) |

Within each row, means for each condition that do not share a subscript are significantly different. Subscripts do not compare between rows. †p < .10, * p < .05, ** p < .01, *** p < .001

# Multiple Comparisons

In the main text, we use subscripts to note significant differences between groups for all of our multiple comparisons, in order to make the tables easier to more easily compare across studies. For the sake of transparency, we report exact t- and p-values in Tables S11-S17 below.

To ensure that the effects were robust, we also analyzed the comparisons using a Holm-Bonferroni correction. The Holm-Bonferroni method orders all p-values in the set from lowest to highest. The first p-value is considered significant if it is smaller than the critical value (in this case α = .050) divided by the number of comparisons (k). The second p-value is significant if it is smaller than α/(k-1), the third if it is smaller than α/(k-2), and so on until stopping at the first comparison to be found as non-significant. For example, for each trust variable in Table S13, we have 6 comparisons. So the smallest p-value had to be smaller than .050/6 = .008, the second smallest had to be smaller than .050/5 = .010, the third .050/4 = .013, and so on. Comparisons that remained significant are indicated with asterisks. Overall, results were not substantially different with the correction applied. Only a few comparisons became non-significant, and only one of these was relevant to our main hypotheses: We predicted that trust would drop in the specific source that made an error. Across all studies we observe this pattern for all sources. A single exception appeared with the Bonferroni-Holm correction in Study 2; the difference between the mainstream error condition and the control condition became non-significant for mainstream trust. However, this drop in significance is likely an overcorrection – this finding was predicted in advance, significant in Study 2 without the Holm-Bonferroni correction, and significant in Studies 1 and 3 even with Holm-Bonferroni correction, and as such is a robust and replicated finding.

To illustrate the size of the effect for key comparisons, we also display Cohen’s d for the comparisons between the control condition and each error condition for each of the primary trust variables across studies in Figure S1. Notably, the largest effects observed are the decrease in trust in the condition where the source in question makes the error, while drops in trust caused by the error of a different source (consistent with the General Loss Model) are somewhat smaller.

## Table S11. Post hoc tests for main effect of source on primary trust measures.

|  | Comparison | *t* | *p* |
| --- | --- | --- | --- |
| Study 1 | Mainstream — Counter-Mainstream | 28.93 | < .001* |
|  | Mainstream — Neutral | 2.57 | < .001* |
|  | Counter-Mainstream — Neutral | -7.22 | < .001* |
|  |  |  |  |
| Study 2 | Mainstream — Counter-Mainstream | 2.76 | < .001* |
|  | Mainstream — Neutral | 3.08 | < .001* |
|  | Counter-Mainstream — Neutral | 0.35 | .732 |
|  |  |  |  |
| Study 3 | Mainstream — Counter-Mainstream | 16.79 | < .001* |
|  | Mainstream — Neutral | 10.96 | < .001* |
|  | Counter-Mainstream — Neutral | -7.37 | < .001* |

Asterisks indicate that the effect remains significant after applying a Holm-Bonferroni correction.

## Table S12. Post hoc tests for main effect of error condition on primary trust measures.

|  | Comparison | *t* | *p* |
| --- | --- | --- | --- |
| Study 1 | Mainstream Error — Counter-Mainstream Error | 1.20 | .232 |
|  | Mainstream Error — Neutral Error | 3.27 | .001* |
|  | Mainstream Error — Control | -3.36 | < .001* |
|  | Counter-Mainstream Error — Neutral Error | 2.05 | .040 |
|  | Counter-Mainstream Error — Control | - 4.55 | < .001* |
|  | Neutral Error — Control | -6.60 | < .001* |
|  |  |  |  |
| Study 2 | Mainstream Error — Counter-Mainstream Error | 2.23 | .026 |
|  | Mainstream Error — Neutral Error | 2.33 | .020 |
|  | Mainstream Error — Control | - 1.79 | .074 |
|  | Counter-Mainstream Error — Neutral Error | 0.12 | .904 |
|  | Counter-Mainstream Error — Control | - 4.01 | < .001* |
|  | Neutral Error — Control | - 4.09 | < .001* |
|  |  |  |  |
| Study 3 | Mainstream Error — Counter-Mainstream Error | 2.29 | .022* |
|  | Mainstream Error — Control | - 10.68 | < .001* |
|  | Counter-Mainstream Error — Control | -12.91 | < .001* |

Asterisks indicate that the effect remains significant after applying a Holm-Bonferroni correction.

## Table S13. Post hoc tests for source × error interaction on primary trust measures.

|  | Source | Comparison | *t* | *p* |
| --- | --- | --- | --- | --- |
| Study 1 | Mainstream | Mainstream Error — Counter-Mainstream Error | -2.51 | .012* |
|  |  | Mainstream Error — Neutral Error | -2.93 | .003* |
|  |  | Mainstream Error — Control | - 2.69 | .007* |
|  |  | Counter-Mainstream Error — Neutral Error | - 0.42 | .672 |
|  |  | Counter-Mainstream Error — Control | - 0.19 | .852 |
|  |  | Neutral Error — Control | 0.24 | .814 |
|  | Counter-Mainstream | Mainstream Error — Counter-Mainstream Error | 4.95 | < .001* |
|  |  | Mainstream Error — Neutral Error | 1.81 | .070 |
|  |  | Mainstream Error — Control | - 0.66 | .511 |
|  |  | Counter-Mainstream Error — Neutral Error | - 3.12 | .002* |
|  |  | Counter-Mainstream Error — Control | - 5.59 | < .001* |
|  |  | Neutral Error — Control | - 2.46 | .014* |
|  | Neutral | Mainstream Error — Counter-Mainstream Error | - 0.19 | .847 |
|  |  | Mainstream Error — Neutral Error | 7.95 | < .001* |
|  |  | Mainstream Error — Control | - 3.61 | < .001* |
|  |  | Counter-Mainstream Error — Neutral Error | 8.13 | < .001* |
|  |  | Counter-Mainstream Error — Control | - 3.41 | < .001* |
|  |  | Neutral Error — Control | - 11.50 | < .001* |
| Study 2 | Mainstream | Mainstream Error — Counter-Mainstream Error | - 1.98 | .049 |
|  |  | Mainstream Error — Neutral Error | - 0.65 | .517 |
|  |  | Mainstream Error — Control | - 2.53 | .011 |
|  |  | Counter-Mainstream Error — Neutral Error | 1.31 | .189 |
|  |  | Counter-Mainstream Error — Control | - 0.59 | .556 |
|  |  | Neutral Error — Control | - 1.88 | .060 |
|  | Counter-Mainstream | Mainstream Error — Counter-Mainstream Error | 4.76 | < .001* |
|  |  | Mainstream Error — Neutral Error | 0.50 | .619 |
|  |  | Mainstream Error — Control | - 0.74 | .459 |
|  |  | Counter-Mainstream Error — Neutral Error | - 4.24 | < .001* |
|  |  | Counter-Mainstream Error — Control | - 5.46 | < .001* |
|  |  | Neutral Error — Control | - 1.23 | .219 |
|  | Neutral | Mainstream Error — Counter-Mainstream Error | 1.54 | .125 |
|  |  | Mainstream Error — Neutral Error | 5.21 | < .001* |
|  |  | Mainstream Error — Control | - 0.34 | .728 |
|  |  | Counter-Mainstream Error — Neutral Error | 3.70 | < .001* |
|  |  | Counter-Mainstream Error — Control | - 1.87 | .062 |
|  |  | Neutral Error — Control | - 5.51 | < .001* |
| Study 3 | Mainstream | Mainstream Error — Counter-Mainstream Error | - 7.79 | < .001* |
|  |  | Mainstream Error — Control | - 13.36 | < .001* |
|  |  | Counter-Mainstream Error — Control | - 5.58 | < .001* |
|  | Counter-Mainstream | Mainstream Error — Counter-Mainstream Error | 10.74 | < .001* |
|  |  | Mainstream Error — Control | - 4.06 | < .001* |
|  |  | Counter-Mainstream Error — Control | - 14.71 | < .001* |
|  | Neutral | Mainstream Error — Counter-Mainstream Error | 2.27 | .023* |
|  |  | Mainstream Error — Control | - 6.36 | < .001* |
|  |  | Counter-Mainstream Error — Control | - 8.59 | < .001* |

Asterisks indicate that the effect remains significant after applying a Holm-Bonferroni correction, based on the number of comparisons within each trust variable (6 comparisons in Studies 1 & 2, 3 comparisons in Study 3).

## Table S14. Post hoc tests for political party × error interaction on primary trust measures.

|  | Party | Comparison | *t* | *p* |
| --- | --- | --- | --- | --- |
| Study 1 | Republican | Mainstream Error — Counter-Mainstream Error | 1.05 | .294 |
|  |  | Mainstream Error — Neutral Error | 2.17 | .031 |
|  |  | Mainstream Error — Control | - 1.32 | .187 |
|  |  | Counter-Mainstream Error — Neutral Error | 1.15 | .250 |
|  |  | Counter-Mainstream Error — Control | - 2.36 | .019 |
|  |  | Neutral Error — Control | - 3.43 | < .001* |
|  | Democrat | Mainstream Error — Counter-Mainstream Error | 0.64 | .524 |
|  |  | Mainstream Error — Neutral Error | 2.45 | .015* |
|  |  | Mainstream Error — Control | - 3.54 | < .001* |
|  |  | Counter-Mainstream Error — Neutral Error | 1.77 | .077 |
|  |  | Counter-Mainstream Error — Control | - 4.14 | < .001* |
|  |  | Neutral Error — Control | - 6.03 | < .001* |
| Study 2 | Republican | Mainstream Error — Counter-Mainstream Error | 1.37 | .172 |
|  |  | Mainstream Error — Neutral Error | 1.73 | .083 |
|  |  | Mainstream Error — Control | - 0.61 | .543 |
|  |  | Counter-Mainstream Error — Neutral Error | 0.38 | .702 |
|  |  | Counter-Mainstream Error — Control | - 1.97 | .049 |
|  |  | Neutral Error — Control | - 2.33 | .020 |
|  | Democrat | Mainstream Error — Counter-Mainstream Error | 1.81 | .070 |
|  |  | Mainstream Error — Neutral Error | 1.56 | .120 |
|  |  | Mainstream Error — Control | - 2.00 | .045 |
|  |  | Counter-Mainstream Error — Neutral Error | - 0.23 | .817 |
|  |  | Counter-Mainstream Error — Control | - 3.78 | < .001* |
|  |  | Neutral Error — Control | - 3.51 | < .001* |
| Study 3 | Republican | Mainstream Error — Counter-Mainstream Error | 0.48 | .630 |
|  |  | Mainstream Error — Control | - 7.76 | < .001* |
|  |  | Counter-Mainstream Error — Control | - 8.17 | < .001* |
|  | Democrat | Mainstream Error — Counter-Mainstream Error | 2.80 | .005* |
|  |  | Mainstream Error — Control | - 7.34 | < .001* |
|  |  | Counter-Mainstream Error — Control | - 10.11 | < .001* |

Asterisks indicate that the effect remains significant after applying a Holm-Bonferroni correction, based on the number of comparisons within each political group (6 comparisons in Studies 1 & 2, 3 comparisons in Study 3).

## Table S15. Post hoc tests for 3-way interaction on primary trust measures.

| **STUDY 2** | | | |
| --- | --- | --- | --- |
| **Mainstream Source** | | | |
| Party | Comparison | *t* | *p* |
| Republican | Mainstream Error — Counter-Mainstream Error | - 0.15 | .877 |
|  | Mainstream Error — Neutral Error | 1.12 | .263 |
|  | Mainstream Error — Control | - 1.21 | .227 |
|  | Counter-Mainstream Error — Neutral Error | 1.29 | .197 |
|  | Counter-Mainstream Error — Control | - 1.07 | .284 |
|  | Neutral Error — Control | - 2.33 | .020 |
| Democrat | Mainstream Error — Counter-Mainstream Error | - 2.76 | .006* |
|  | Mainstream Error — Neutral Error | - 2.18 | .029 |
|  | Mainstream Error — Control | - 2.45 | .014 |
|  | Counter-Mainstream Error — Neutral Error | 0.54 | .588 |
|  | Counter-Mainstream Error — Control | 0.30 | .763 |
|  | Neutral Error — Control | - 2.45 | .808 |
| **Counter-Mainstream Source** | | | |
| Republican | Mainstream Error — Counter-Mainstream Error | 2.10 | .036 |
|  | Mainstream Error — Neutral Error | 0.49 | .624 |
|  | Mainstream Error — Control | 0.13 | .898 |
|  | Counter-Mainstream Error — Neutral Error | - 1.61 | .107 |
|  | Counter-Mainstream Error — Control | - 1.95 | .052 |
|  | Neutral Error — Control | - 0.36 | .721 |
| Democrat | Mainstream Error — Counter-Mainstream Error | 4.77 | < .001* |
|  | Mainstream Error — Neutral Error | 0.20 | .842 |
|  | Mainstream Error — Control | - 1.26 | .209 |
|  | Counter-Mainstream Error — Neutral Error | - 4.48 | < .001* |
|  | Counter-Mainstream Error — Control | -5.97 | < .001* |
|  | Neutral Error — Control | - 1.43 | .153 |
| **Neutral Source** | | | |
| Republican | Mainstream Error — Counter-Mainstream Error | 0.75 | .453 |
|  | Mainstream Error — Neutral Error | 2.03 | .043 |
|  | Mainstream Error — Control | - 0.18 | .855 |
|  | Counter-Mainstream Error — Neutral Error | 1.30 | .195 |
|  | Counter-Mainstream Error — Control | - 0.93 | .353 |
|  | Neutral Error — Control | - 2.19 | .029 |
| Democrat | Mainstream Error — Counter-Mainstream Error | 1.46 | .145 |
|  | Mainstream Error — Neutral Error | 5.50 | < .001* |
|  | Mainstream Error — Control | -0.32 | .751 |
|  | Counter-Mainstream Error — Neutral Error | 4.04 | < .001* |
|  | Counter-Mainstream Error — Control | - 1.76 | .079 |
|  | Neutral Error — Control | - 5.77 | < .001* |
| **STUDY 3** | | | |
| **Mainstream Source** | | | |
| Party | Comparison | *t* | *p* |
| Republican | Mainstream Error — Counter-Mainstream Error | - 4.59 | < .001* |
|  | Mainstream Error — Control | - 7.84 | < .001* |
|  | Counter-Mainstream Error — Control | - 3.20 | .001* |
| Democrat | Mainstream Error — Counter-Mainstream Error | - 6.45 | < .001* |
|  | Mainstream Error — Control | -11.07 | < .001* |
|  | Counter-Mainstream Error — Control | -4.71 | < .001* |
| **Counter-Mainstream Source** | | | |
| Republican | Mainstream Error — Counter-Mainstream Error | 5.39 | < .001* |
|  | Mainstream Error — Control | - 3.83 | < .001* |
|  | Counter-Mainstream Error — Control | - 9.17 | < .001* |
| Democrat | Mainstream Error — Counter-Mainstream Error | 9.86 | < .001* |
|  | Mainstream Error — Control | - 1.90 | .057 |
|  | Counter-Mainstream Error — Control | - 11.67 | < .001* |
| **Neutral Source** | | | |
| Republican | Mainstream Error — Counter-Mainstream Error | 0.31 | .750 |
|  | Mainstream Error — Control | - 5.69 | < .001* |
|  | Counter-Mainstream Error — Control | - 5.94 | < .001** |
| Democrat | Mainstream Error — Counter-Mainstream Error | 2.95 | .003* |
|  | Mainstream Error — Control | - 3.29 | .001* |
|  | Counter-Mainstream Error — Control | - 6.21 | < .001* |

Asterisks indicate that the effect remains significant after applying a Holm-Bonferroni correction, based on the number of comparisons within each political group (6 comparisons in Studies 1 & 2, 3 comparisons in Study 3).

## Table S16. Post hoc tests for perceptions of politicization, Study 2.

| **Error Condition** | | | | | |
| --- | --- | --- | --- | --- | --- |
| Comparison | | *t* | | *p* | |
| Mainstream Error — Counter-Mainstream Error | | 1.57 | | .119 | |
| Mainstream Error — Neutral Error | | 0.84 | | .401 | |
| Counter-Mainstream Error — Neutral Error | | 0.71 | | .478 | |
| **Political Party × Error Condition** | | | | | |
| Source | Comparison | | *t* | | *p* |
| Republican | Mainstream Error — Counter-Mainstream Error | | 0.36 | | .719 |
|  | Mainstream Error — Neutral Error | | 0.01 | | .992 |
|  | Counter-Mainstream Error — Neutral Error | | - 0.35 | | .724 |
| Democrat | Mainstream Error — Counter-Mainstream Error | | 1.92 | | .055 |
|  | Mainstream Error — Neutral Error | | 1.23 | | .219 |
|  | Counter-Mainstream Error — Neutral Error | | - 0.66 | | .508 |

## Table S17. Post hoc tests for perceptions of politicization, Study 3.

| **Source** | | | | | | | | |
| --- | --- | --- | --- | --- | --- | --- | --- | --- |
| Comparison | | | *t* | | | *p* | | |
| Mainstream — Counter-Mainstream | | | - 8.63 | | | < .001* | | |
| Mainstream — Neutral | | | 4.29 | | | < .001* | | |
| Counter-Mainstream — Neutral | | | 15.02 | | | < .001* | | |
| **Error Condition** | | | | | | | | |
| Comparison | | | *t* | | | *p* | | |
| Mainstream Error — Counter-Mainstream Error | | | -1.30 | | | .196 | | |
| **Source × Error Condition** | | | | | | | | |
| Source | | Comparison | | *t* | | | *p* | |
| Mainstream | | Mainstream Error — Counter-Mainstream Error | | 3.60 | | | < .001* | |
| Counter-Mainstream | | Mainstream Error — Counter-Mainstream Error | | - 1.66 | | | .097 | |
| Neutral | | Mainstream Error — Counter-Mainstream Error | | 0.84 | | | .403 | |
| **Political Party × Error Condition** | | | | | | | | |
| Source | Comparison | | | | *t* | | | *p* |
| Republican | Mainstream Error — Counter-Mainstream Error | | | | 1.30 | | | .117 |
| Democrat | Mainstream Error — Counter-Mainstream Error | | | | 0.51 | | | .605 |

Asterisks indicate that the effect remains significant after applying a Holm-Bonferroni correction.

## Figure S1


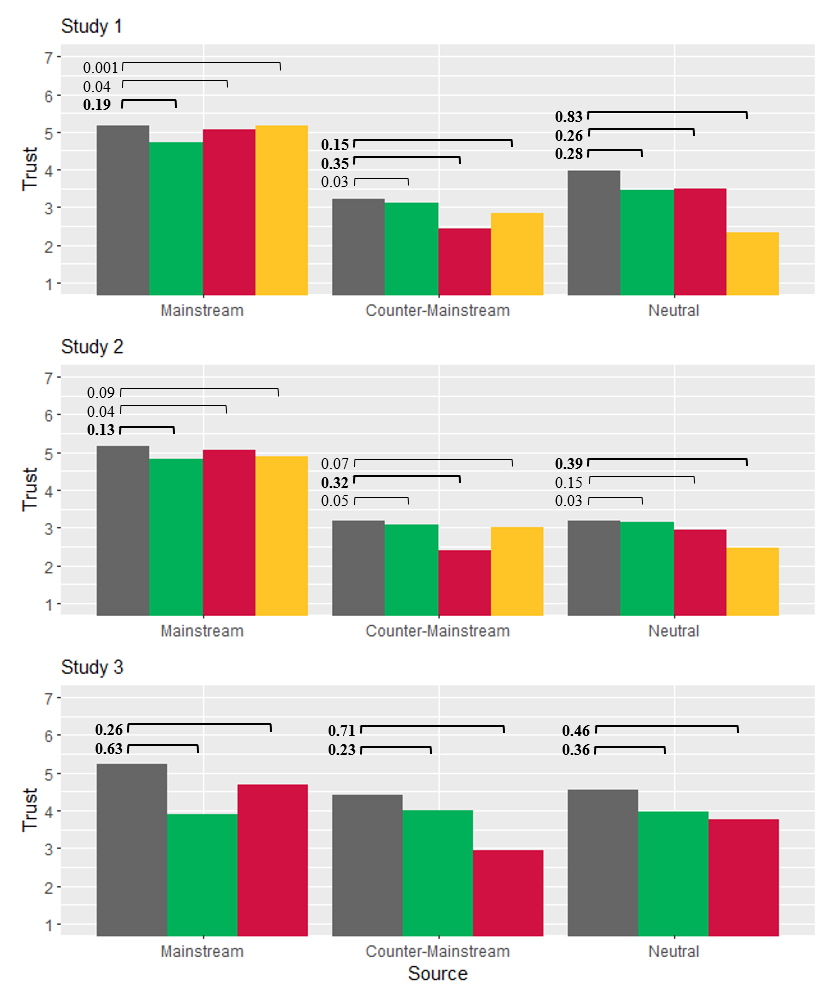
*Effect sizes (Cohen’s d) for comparisons between control condition and error conditions for primary trust measures.*


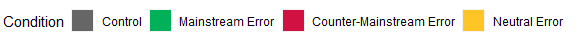


*Note.* Bolded effects are statistically significant.

# Additional Sources

As an exploratory analysis, we were also interested in whether people would shift their trust in a number of additional sources beyond the target sources (e.g. federal government, tabloids, local church) in response to our manipulation. To examine this we ran separate ANOVAs for each additional variable, applying a Holm-Bonferroni correction so that each main effect, interaction, and multiple comparison accounted for the number of additional trust variables in the study. See Table S18 for full results.

## Table S18. Main effects and interactions with estimated marginal means and standard errors for additional sources.

| Study 1 | | | | | | | | | | |
| --- | --- | --- | --- | --- | --- | --- | --- | --- | --- | --- |
|  | Main Effect  Political Party | Republican | Democrat | Main Effect Manipulation | Mainstream Error | Counter-Mainstream Error | Neutral Error | Control | Political Party × Manipulation |  |
| US Federal Government | *F*(1, 779) = 115.68,  η_p_^2^ = .13*** | 3.25  (0.08) | 4.42  (0.08) | *F*(3, 779) = 0.21,  η_p_^2^ = .001 | 3.87_a_  (0.11) | 3.79_a_  (0.11) | 3.89_a_  (0.11) | 3.79_a_  (0.11) | *F*(3, 779) = 0.33,  η_p_^2^ = .001 |  |
| Academic Scientists | *F*(1, 779) = 228.76,  η_p_^2^ = .23*** | 4.28  (0.07) | 5.72  (0.07) | *F*(3, 779) = .09,  η_p_^2^ < .001 | 4.96_a_  (0.10) | 5.01_a_  (0.10) | 5.02_a_  (0.10) | 4.99_a_  (0.10) | *F*(3, 779) = 0.37,  η_p_^2^ = .001 |  |
| Washington Post | *F*(1, 778) = 174.62,  η_p_^2^ = .18*** | 3.05  (0.08) | 4.57  (0.08) | *F*(3, 778) = 0.87,  η_p_^2^ = .003 | 3.89_a_  (0.11) | 3.70_a_  (0.11) | 3.73_a_  (0.12) | 3.91_a_  (0.12) | *F*(3, 778) = 0.64,  η_p_^2^ = .002 |  |
| Reuters | *F*(1, 772) = 84.14,  η_p_^2^ = .10*** | 3.43  (0.09) | 4.52  (0.08) | *F*(3, 772) = 1.37,  η_p_^2^ = .01 | 4.01_ab_  (0.12) | 3.99_ab_  (0.12) | 3.78_a_  (0.12) | 4.12_b_  (0.12) | *F*(3, 772) = 0.38,  η_p_^2^ = .001 |  |
| Wall Street Journal | *F*(1, 778) = 20.08,  η_p_^2^ = .03*** | 3.66  (0.09) | 4.19  (0.08) | *F*(3, 778) = 0.53,  η_p_^2^ = .002 | 3.96_a_  (0.12) | 3.95_a_  (0.12) | 3.80_a_  (0.12) | 3.99_a_  (0.12) | *F*(3, 778) = 1.23,  η_p_^2^ = .01 |  |
| Mainstream media in general | *F*(1, 779) = 175.50,  η_p_^2^ = .18*** | 2.94  (0.08) | 4.34  (0.07) | *F*(3, 779) = 0.99,  η_p_^2^ = .004 | 3.74_a_  (0.11) | 3.50_a_  (0.11) | 3.62_a_  (0.11) | 3.70_a_  (0.11) | *F*(3, 779) = 0.78,  η_p_^2^ = .003 |  |
| Occupy Democrats | *F*(1, 760) = 240.70,  η_p_^2^ = .24*** | 1.74  (0.07) | 3.32  (0.07) | *F*(3, 760) = 0.44,  η_p_^2^ = .002 | 2.59_a_  (0.10) | 2.58_a_  (0.10) | 2.47_a_  (0.10) | 2.47_a_  (0.10) | *F*(3, 760) = 0.20,  η_p_^2^ = .001 |  |
| Breitbart | *F*(1, 760) = 159.79,  η_p_^2^ = .17*** | 3.15  (0.08) | 1.81  (0.07) | *F*(3, 760) = 1.99,  η_p_^2^ = .01 | 2.50_ab_  (0.10) | 2.66_a_  (0.11) | 2.29_b_  (0.11) | 2.47_ab_  (0.11) | *F*(3, 760) = 1.05,  η_p_^2^ = .004 |  |
| Tabloid Magazines | *F*(1, 779) = 2.09,  η_p_^2^ = .003 | 1.76  (0.06) | 1.64  (0.06) | *F*(3, 779) = 0.05,  η_p_^2^ < .001 | 1.72_a_  (0.08) | 1.70_a_  (0.08) | 1.69_a_  (0.08) | 1.68_a_  (0.08) | *F*(3, 779) = 2.46,  η_p_^2^ = .009† |  |
| Counter-mainstream media in general | *F*(1, 776) = 54.96,  η_p_^2^ = .07*** | 3.35  (0.08) | 2.58  (0.07) | *F*(3, 776) = 0.77,  η_p_^2^ = .003 | 2.95_a_  (0.10) | 3.09_a_  (0.10) | 2.87_a_  (0.10) | 2.97_a_  (0.10) | *F*(3, 776) = 0.10,  η_p_^2^ < .001 |  |
| Fox News | *F*(1, 779) = 556.31,  η_p_^2^ = .42*** | 4.21  (0.08) | 1.75  (0.07) | *F*(3, 779) = 0.99,  η_p_^2^ = .004 | 2.98_a_  (0.10) | 3.07_a_  (0.10) | 2.84_a_  (0.11) | 3.04_a_  (0.11) | *F*(3, 779) = 0.75,  η_p_^2^ = .003 |  |
| Your local community organizations | *F*(1, 776) = 0.14,  η_p_^2^ < .001 | 4.12  (0.07) | 4.08  (0.07) | *F*(3, 776) = 0.38,  η_p_^2^ = .001 | 4.16_a_  (0.10) | 4.02_a_  (0.10) | 4.10_a_  (0.10) | 4.13_a_  (0.10) | *F*(3, 776) = 0.72,  η_p_^2^ = .003 |  |
| Sports blogs in general | *F*(1, 772) = 0.97,  η_p_^2^ = .001 | 3.16  (0.08) | 3.06  (0.07) | *F*(3, 772) = 6.96,  η_p_^2^ = .03*** | 3.13_a_  (0.11) | 3.17_a_  (0.17) | 2.74_b_  (0.11) | 3.41_a_  (0.11) | *F*(3, 772) = 1.52,  η_p_^2^ = .01 |  |
| Your local church | *F*(1, 764) = 216.60,  η_p_^2^ = .22*** | 4.46  (0.09) | 2.64  (0.09) | *F*(3, 764) = 1.05,  η_p_^2^ = .004 | 3.54_a_  (0.12) | 3.58_a_  (0.12) | 3.39_a_  (0.13) | 3.70_a_  (0.12) | *F*(3, 764) = 0.09,  η_p_^2^ < .001 |  |
| Your favorite YouTubers | *F*(1, 768) = 0.34,  η_p_^2^ < .001 | 3.50  (0.08) | 3.43  (0.08) | *F*(3, 768) = 0.62,  η_p_^2^ = .002 | 3.41_a_  (0.11) | 3.41_a_  (0.11) | 3.44_a_  (0.11) | 3.60_a_  (0.11) | *F*(3, 768) = 0.51,  η_p_^2^ = .002 |  |
| Your favorite podcasts | *F*(1, 764) = 0.28,  η_p_^2^ < .001 | 4.03  (0.08) | 3.97  (0.07) | *F*(3, 764) = 0.59,  η_p_^2^ = .002 | 3.93_a_  (0.11) | 3.97_a_  (0.11) | 3.98_a_  (0.11) | 4.12_a_  (0.11) | *F*(3, 764) = 0.11,  η_p_^2^ < .001 |  |
| Your social media feed | *F*(1, 774) = 0.14,  η_p_^2^ < .001 | 3.22  (0.08) | 3.26  (0.07) | *F*(3, 774) = 0.24,  η_p_^2^ = .001 | 3.23_a_  (0.11) | 3.19_a_  (0.11) | 3.22_a_  (0.11) | 3.31_a_  (0.11) | *F*(3, 774) = 2.25,  η_p_^2^ = .01† |  |
| Your friends and family | *F*(1, 779) = 21.99,  η_p_^2^ = .03*** | 5.02  (0.07) | 4.56  (0.07) | *F*(3, 779) = 4.67,  η_p_^2^ = .02** | 4.64_a_  (0.10) | 4.72_a_  (0.10) | 4.70_a_  (0.10) | 5.10_b_  (0.10) | *F*(3, 779) = 0.56,  η_p_^2^ = .002 |  |
| Study 2 | | | | | | | | | | |
|  | Main Effect  Political Party | Republican | Democrat | Main Effect Manipulation | Mainstream Error | Counter-Mainstream Error | Neutral Error | Control | Political Party × Manipulation |  |
| US Federal Government | *F*(1, 934) = 255.76,  η_p_^2^ = .22*** | 3.25  (0.07) | 4.80  (0.07) | *F*(3, 934) = 1.46,  η_p_^2^ = .01 | 4.11_a_  (0.10) | 4.03_a_  (0.10) | 3.86_a_  (0.10) | 4.10_a_  (0.10) | *F*(3, 934) = 3.41,  η_p_^2^ = .01* |  |
| Academic Scientists | *F*(1, 934) = 299.29,  η_p_^2^ = .24*** | 4.30  (0.06) | 5.80  (0.06) | *F*(3, 934) = 1.10,  η_p_^2^ = .004 | 5.17_a_  (0.09) | 4.98_a_  (0.09) | 4.98_a_  (0.09) | 5.08_a_  (0.09) | *F*(3, 934) = 1.13,  η_p_^2^ = .004 |  |
| Washington Post | *F*(1, 933) = 282.96,  η_p_^2^ = .23*** | 2.94  (0.07) | 4.62  (0.07) | *F*(3, 933) = 2.82,  η_p_^2^ = .01* | 3.91_a_  (0.10) | 3.73_ab_  (0.10) | 3.56_b_  (0.10) | 3.91_a_  (0.10) | *F*(3, 933) = 0.74,  η_p_^2^ = .002 |  |
| Reuters | *F*(1, 916) = 206.72,  η_p_^2^ = .18*** | 3.10  (0.08) | 4.59  (0.07) | *F*(3, 916) = 2.69,  η_p_^2^ = .01* | 3.99_a_  (0.10) | 3.79_ab_  (0.10) | 3.63_b_  (0.10) | 3.98_a_  (0.11) | *F*(3, 916) = 0.66,  η_p_^2^ = .002 |  |
| Wall Street Journal | *F*(1, 933) = 81.86,  η_p_^2^ = .08*** | 3.34  (0.08) | 4.26  (0.07) | *F*(3, 933) = 3.12,  η_p_^2^ = .01* | 3.91_a_  (0.10) | 3.77_ab_  (0.10) | 3.56_b_  (0.10) | 3.96_a_  (0.10) | *F*(3, 933) = 0.58,  η_p_^2^ = .002 |  |
| Mainstream media in general | *F*(1, 934) = 279.33,  η_p_^2^ = .23*** | 2.78  (0.07) | 4.37  (0.06) | *F*(3, 934) = 3.30,  η_p_^2^ = .01* | 3.68_a_  (0.10) | 3.59_ab_  (0.09) | 3.32_b_  (0.10) | 3.70_a_  (0.10) | *F*(3, 934) = 0.19,  η_p_^2^ = .001 |  |
| Occupy Democrats | *F*(1, 921) = 338.56,  η_p_^2^ = .27*** | 1.80  (0.07) | 3.53  (0.06) | *F*(3, 921) = 0.48,  η_p_^2^ = .002 | 2.71_a_  (0.09) | 2.74_a_  (0.09) | 2.63_a_  (0.09) | 2.60_a_  (0.10) | *F*(3, 921) = 0.82,  η_p_^2^ = .003 |  |
| Breitbart | *F*(1, 896) = 45.93,  η_p_^2^ = .05*** | 2.97  (0.08) | 2.24  (0.07) | *F*(3, 896) = 0.92,  η_p_^2^ = .003 | 2.72_a_  (0.11) | 2.50_a_  (0.11) | 2.54_a_  (0.11) | 2.66_a_  (0.11) | *F*(3, 896) = 0.81,  η_p_^2^ = .003 |  |
| Tabloid Magazines | *F*(1, 934) = 1.15,  η_p_^2^ = .001 | 1.77  (0.07) | 1.87  (0.06) | *F*(3, 934) = 0.23,  η_p_^2^ = .001 | 1.86_a_  (0.09) | 1.83_a_  (0.09) | 1.76_a_  (0.09) | 1.82_a_  (0.09) | *F*(3, 934) = 0.73,  η_p_^2^ = .002 |  |
| Counter-mainstream media in general | *F*(1, 934) = 73.79,  η_p_^2^ = .07*** | 3.29  (0.07) | 2.43  (0.07) | *F*(3, 934) = 2.35,  η_p_^2^ = .01† | 2.94_a_  (0.10) | 2.64_b_  (0.10) | 2.92_a_  (0.10) | 2.96_a_  (0.10) | *F*(3, 934) = 0.51,  η_p_^2^ = .002 |  |
| Fox News | *F*(1, 933) = 329.29,  η_p_^2^ = .26*** | 4.05  (0.08) | 2.12  (0.07) | *F*(3, 933) = 1.53,  η_p_^2^ = .01 | 3.18_a_  (0.11) | 2.92_a_  (0.11) | 3.05_a_  (0.11) | 3.20_a_  (0.11) | *F*(3, 933) = 1.68,  η_p_^2^ = .01 |  |
| Your local community organizations | *F*(1, 934) = 4.43,  η_p_^2^ = .01* | 3.51  (0.06) | 3.67  (0.05) | *F*(3, 934) = 1.18,  η_p_^2^ = .004 | 3.64_a_  (0.08) | 3.46_a_  (0.08) | 3.62_a_  (0.08) | 3.64_a_  (0.08) | *F*(3, 934) = 0.51,  η_p_^2^ = .002 |  |
| Sports blogs in general | *F*(1, 934) = 2.17,  η_p_^2^ = .002 | 2.98  (0.06) | 3.11  (0.06) | *F*(3, 934) = 3.00,  η_p_^2^ = .01* | 3.16_ab_  (0.09) | 2.94_bc_  (0.09) | 2.89_c_  (0.09) | 3.19_a_  (0.09) | *F*(3, 934) = 1.41,  η_p_^2^ = .01* |  |
| Your local church | *F*(1, 928) = 86.19,  η_p_^2^ = .09* | 3.88  (0.08) | 2.82  (0.08) | *F*(3, 928) = 0.62,  η_p_^2^ = .002 | 3.33_a_  (0.11) | 3.23_a_  (0.11) | 3.43_a_  (0.11) | 3.41_a_  (0.12) | *F*(3, 928) = 3.60,  η_p_^2^ = .01* |  |
| Your favorite YouTubers | *F*(1, 928) = 2.02,  η_p_^2^ = .002 | 3.21  (0.08) | 3.06  (0.07) | *F*(3, 928) = 0.29,  η_p_^2^ = .001 | 3.17_a_  (0.10) | 3.09_a_  (0.10) | 3.09_a_  (0.11) | 3.20_a_  (0.11) | *F*(3, 928) = 1.23,  η_p_^2^ = .004 |  |
| Your favorite podcasts | *F*(1, 926) = 0.69,  η_p_^2^ = .001 | 3.59  (0.08) | 3.51  (0.07) | *F*(3, 926) = 1.91,  η_p_^2^ = .01 | 3.67_a_  (0.10) | 3.39_a_  (0.10) | 3.47_a_  (0.10) | 3.67_a_  (0.10) | *F*(3, 926) = 2.47,  η_p_^2^ = .01† |  |
| Your social media feed | *F*(1, 933) = 1.78,  η_p_^2^ = .002 | 3.00  (0.08) | 3.14  (0.07) | *F*(3, 933) = 2.71,  η_p_^2^ = .01* | 3.28_a_  (0.10) | 2.88_b_  (0.10) | 3.08_ab_  (0.10) | 3.04_ab_  (0.10) | *F*(3, 933) = 0.25,  η_p_^2^ = .001 |  |
| Your friends and family | *F*(1, 934) = 4.80,  η_p_^2^ = .01* | 4.65  (0.07) | 4.45  (0.06) | *F*(3, 934) = 1.87,  η_p_^2^ = .01 | 4.62_a_  (0.09) | 4.36_b_  (0.09) | 4.60_ab_  (0.09) | 4.61_ab_  (0.09) | *F*(3, 934) = 1.41,  η_p_^2^ = .01 |  |
| Healthcare Professionals/  Doctors | *F*(1, 934) = 153.44,  η_p_^2^ = .14* | 4.92  (0.06) | 5.91  (005) | *F*(3, 934) = 1.39,  η_p_^2^ = .004 | 5.51_a_  (0.08) | 5.30_a_  (0.08) | 5.37_a_  (0.08) | 5.47_a_  (0.08) | *F*(3, 934) = 1.37,  η_p_^2^ = .004 |  |
| Study 3 | | | | | | | | | | |
|  | Main Effect  Political Party | Republican | Democrat | Main Effect Manipulation | Mainstream Error | Counter-Mainstream Error | Neutral Error | Control | Political Party × Manipulation |  |
| US Federal Government | *F*(1, 1216) = 163.20,  η_p_^2^ = .12*** | 3.30  (0.06) | 4.44  (0.06) | *F*(2, 1216) = 2.35,  η_p_^2^ = .004† | 3.91_ab_  (0.08) | 3.74_a_  (0.08) | — | 3.97_b_  (0.08) | *F*(2, 1216) = 0.33,  η_p_^2^ = .001 |  |
| Academic Scientists | *F*(1, 1214) = 163.20,  η_p_^2^ = .22*** | 4.27  (0.06) | 5.70  (0.05) | *F*(2, 1214) = 2.24,  η_p_^2^ = .004 | 5.01_ab_  (0.07) | 4.88_a_  (0.07) | — | 5.07_b_  (0.07) | *F*(2, 1214) = 0.78,  η_p_^2^ = .001 |  |
| Washington Post | *F*(1, 1207) = 240.44,  η_p_^2^ = .17*** | 3.15  (0.06) | 4.54  (0.06) | *F*(2, 1207) = 2.68,  η_p_^2^ = .04† | 3.86_ab_  (0.08) | 3.71_a_  (0.08) | — | 3.97_b_  (0.08) | *F*(2, 1207) = 1.02,  η_p_^2^ = .002 |  |
| Reuters | *F*(1, 1181) = 163.10,  η_p_^2^ = .12*** | 3.43  (0.07) | 4.65  (0.07) | *F*(2, 1181) = 1.41,  η_p_^2^ = .002 | 4.02_a_  (0.08) | 3.96_a_  (0.08) | — | 4.15_a_  (0.08) | *F*(2, 1181) = 0.80,  η_p_^2^ = .001 |  |
| Wall Street Journal | *F*(1, 1211) = 38.73,  η_p_^2^ = .03*** | 3.69  (0.07) | 4.26  (0.06) | *F*(1, 1211) = 3.76,  η_p_^2^ = .01* | 3.98_ab_  (0.08) | 3.82_a_  (0.08) | — | 4.13_b_  (0.08) | *F*(2, 1211) = 2.44,  η_p_^2^ = .004† |  |
| Mainstream media in general | *F*(1, 1216) = 221.48,  η_p_^2^ = .15*** | 2.96  (0.06) | 4.23  (0.06) | *F*(2, 1216) = 3.78,  η_p_^2^ = .01* | 3.66_a_  (0.07) | 3.43_b_  (0.07) | — | 3.70_a_  (0.07) | *F*(2, 1216) = 1.27,  η_p_^2^ = .002 |  |
| Occupy Democrats | *F*(1, 1180) = 387.70,  η_p_^2^ = .24*** | 1.94  (0.06) | 3.61  (0.06) | *F*(2, 1180) = 5.67,  η_p_^2^ = .01** | 2.91_a_  (0.07) | 2.58_b_  (0.07) | — | 2.84_a_  (0.07) | *F*(2, 1180) = 1.13,  η_p_^2^ = .002 |  |
| Breitbart | *F*(1, 1152) = 122.10,  η_p_^2^ = .10*** | 3.18  (0.07) | 2.15  (0.06) | *F*(2, 1152) = 0.59,  η_p_^2^ = .001 | 2.69_a_  (0.08) | 2.59_a_  (0.08) | — | 2.71_a_  (0.08) | *F*(2, 1152) = 3.56,  η_p_^2^ = .01* |  |
| Tabloid Magazines | *F*(1, 1207) = 0.09,  η_p_^2^ < .001 | 1.89  (0.06) | 1.86  (0.06) | *F*(2, 1207) = 1.60,  η_p_^2^ = .003 | 1.95_a_  (0.07) | 1.78_a_  (0.07) | — | 1.89_a_  (0.07) | *F*(2, 1207) = 2.88,  η_p_^2^ = .01† |  |
| Counter-mainstream media in general | *F*(1, 1207) = 23.77,  η_p_^2^ = .02*** | 3.37  (0.06) | 2.94  (0.06) | *F*(1, 1207) = 2.84,  η_p_^2^ = .01† | 3.16_ab_  (0.08) | 3.03_a_  (0.08) | — | 3.28_b_  (0.08) | *F*(2, 1207) = 2.27,  η_p_^2^ = .004 |  |
| Fox News | *F*(1, 1213) = 410.37,  η_p_^2^ = .25*** | 3.92  (0.07) | 2.05  (0.07) | *F*(2, 1213) = 4.53,  η_p_^2^ = .01* | 3.13_a_  (0.08) | 2.80_b_  (0.08) | — | 3.04_a_  (0.08) | *F*(2, 1213) = 3.66,  η_p_^2^ = .01* |  |
| Your local community organizations | *F*(1, 1213) = 13.71,  η_p_^2^ = .01*** | 4.07  (0.06) | 4.36  (0.04) | *F*(2, 1213) = 6.62,  η_p_^2^ = .01** | 4.24_a_  (0.07) | 4.03_b_  (0.07) | — | 4.37_a_  (0.07) | *F*(2, 1213) = 0.87,  η_p_^2^ = .001 |  |
| Sports blogs in general | *F*(1, 1208) = 1.38,  η_p_^2^ = .001 | 3.34  (0.06) | 3.44  (0.06) | *F*(2, 1208) = 8.67,  η_p_^2^ = .01*** | 3.36_a_  (0.07) | 3.19_a_  (0.08) | — | 3.63_b_  (0.08) | *F*(2, 1208) = 1.11,  η_p_^2^ = .002 |  |
| Your local church | *F*(1, 1197) = 169.19,  η_p_^2^ = .12*** | 4.29  (0.08) | 2.91  (0.07) | *F*(2, 1197) = 0.44,  η_p_^2^ = .001 | 3.61_a_  (0.09) | 3.54_a_  (0.09) | — | 3.66_a_  (0.09) | *F*(2, 1197) = 1.35,  η_p_^2^ = .002 |  |
| Your favorite YouTubers | *F*(1, 1204) = 1.79,  η_p_^2^ = .001 | 3.55  (0.07) | 3.42  (0.06) | *F*(2, 1204) = 2.71,  η_p_^2^ = .004† | 3.47_ab_  (0.08) | 3.36_a_  (0.08) | — | 3.62_b_  (0.08) | (2, 1204) = 1.90,  η_p_^2^ = .003 |  |
| Your favorite podcasts | *F*(1, 1192) = 0.09,  η_p_^2^ < .001 | 4.03  (0.06) | 4.06  (0.06) | *F*(2, 1192) = 1.18,  η_p_^2^ = .002 | 4.03_a_  (0.08) | 3.98_a_  (0.08) | — | 4.14_a_  (0.08) | *F*(2, 1192) = 2.23,  η_p_^2^ = .004 |  |
| Your social media feed | *F*(1, 1212) = 0.24,  η_p_^2^ < .001 | 3.25  (0.06) | 3.20  (0.06) | *F*(2, 1212) = 3.94,  η_p_^2^ = .01* | 3.24_ab_  (0.07) | 3.07_a_  (0.07) | — | 3.36_b_  (0.08) | *F*(2, 1212) = 6.01,  η_p_^2^ = .01** |  |
| Your friends and family | *F*(1, 1216) = 27.24,  η_p_^2^ = .02*** | 5.11  (0.06) | 4.71  (0.05) | *F*(2, 1216) = 1.37,  η_p_^2^ = .002 | 4.93_a_  (0.07) | 4.82_a_  (0.07) | — | 4.97_a_  (0.07) | *F*(2, 1216) = 2.77,  η_p_^2^ = .01† |  |
| Healthcare Professionals/  Doctors | *F*(1, 1216) = 123.52,  η_p_^2^ = .09*** | 4.80  (0.05) | 5.62  (0.05) | *F*(2, 1216) = 1.64,  η_p_^2^ = .003 | 5.23_a_  (0.06) | 5.12_a_  (0.06) | — | 5.28_a_  (0.06) | *F*(2, 1216) = 0.80,  η_p_^2^ = .001 |  |
| Food and Drug Administration (S3 Only) | *F*(1, 1216) = 195.21,  η_p_^2^ = .14*** | 3.90  (0.06) | 5.12  (0.06) | *F*(2, 1216) = 1.12,  η_p_^2^ = .002 | 4.45_a_  (0.08) | 4.47_a_  (0.08) | — | 4.60_a_  (0.08) | *F*(2, 1216) = 0.10,  η_p_^2^ < .001 |  |

Within each row, means for each condition that do not share a subscript are significantly different. Subscripts do not compare between rows. †p < .10, * p < .05, ** p < .01, *** p < .001

# Correlations Split by Political Party

In Tables S19-21 we report the correlational analyses in each study, looking at Republicans and Democrats separately. Overall, results do not differ substantially across political groups with one main exception: Republicans consistently report a positive relationship between trust in the mainstream source and trust in the counter-mainstream source. Democrats, however, in line with the aggregate analyses reporting in the main text, sometimes report a positive relationship (Study 3), but other times report a negative relationship (Studies 1 and 2).

## Table S19. Correlations between trust measures split by political party, Study 1

| Republican | | | | |  |
| --- | --- | --- | --- | --- | --- |
|  | 1 | 2 | 3 | 4 |  |
| 1 Mainstream Trust | — | — | — | — |  |
| 2 Counter-Mainstream Trust | .11* | — | — | — |  |
| 3 Neutral Trust | .16** | .48*** | — | — |  |
| Democrat | | | | | |
|  | | 1 | 2 | 3 | 4 |
| 1 Mainstream Trust | | — | — | — | — |
| 2 Counter-Mainstream Trust | | -.10* | — | — | — |
| 3 Neutral Trust | | .04 | .32*** | — | — |

†p < .10, * p < .05, ** p < .01, *** p < .001

## Table S20. Correlations between perceived politicization of CDC and trust measures split by political party, Study 2

| Republican | | | | |  |
| --- | --- | --- | --- | --- | --- |
|  | 1 | 2 | 3 | 4 |  |
| 1 Mainstream Trust | — | — | — | — |  |
| 2 Counter-Mainstream Trust | .02 | — | — | — |  |
| 3 Neutral Trust | .10* | .62**** | — | — |  |
| 4 Mainstream Politicization | -.56*** | .31*** | .17** | — |  |
| Democrat | | | | | |
|  | | 1 | 2 | 3 | 4 |
| 1 Mainstream Trust | | — | — | — | — |
| 2 Counter-Mainstream Trust | | -.16*** | — | — | — |
| 3 Neutral Trust | | -.07 | .55*** | — | — |
| 4 Mainstream Politicization | | -.41*** | .40*** | .34*** | — |

†p < .10, * p < .05, ** p < .01, *** p < .001

## Table S21. Correlations between perceived politicization of sources and trust measures split by political party, Study 3.

| Republican | | | | | | | |
| --- | --- | --- | --- | --- | --- | --- | --- |
|  | 1 | 2 | 3 | 4 | 5 | 6 |  |
| 1 Mainstream Trust | — | — | — | — | — | — |  |
| 2 Counter Trust | .37** | — | — | — | — | — |  |
| 3 Neutral Trust | .35*** | .53*** | — | — | — | — |  |
| 4 Mainstream Politicization | -.23*** | .13* | .06 | — | — | — |  |
| 5 Counter Politicization | .06 | -.10* | .002 | .43*** | — | — |  |
| 6 Neutral Politicization | .12* | .03 | -.10* | .43*** | .57*** | — |  |
| Democrat | | | | | | | |
|  | | 1 | 2 | 3 | 4 | 5 | 6 |
| 1 Mainstream Trust | | — | — | — | — | — | — |
| 2 Counter Trust | | .15*** | — | — | — | — | — |
| 3 Neutral Trust | | .29*** | .42*** | — | — | — | — |
| 4 Mainstream Politicization | | -.26*** | .24*** | .09† | — | — | — |
| 5 Counter Politicization | | .10* | -.18*** | .02 | .23*** | — | — |
| 6 Neutral Politicization | | -.04 | .14** | -.04 | .38*** | .39*** | — |

†p < .10, * p < .05, ** p < .01, *** p < .001
