# Supplementary material for: Is trust a zero-sum game? What happens when institutional sources get it wrong
Source: PLoS One. 2025 Apr 22;20(4):e0321743. doi: 10.1371/journal.pone.0321743 (PMC12013948; doi:10.1371/journal.pone.0321743)
Supplement: Attachment — Submitted filename: Is Trust a Zero-Sum Game Response Letter August 07 2024.docx [file pone.0321743.s002.docx]

To the team at PLOS One:

We have responded to your instructions below:

1. *Please complete all items on the Human Participants Research Checklist that are relevant for your submission, by following this link:* [*http://journals.plos.org/plosone/s/file?id=dc11/PLOSOne_Human_Subjects_Research_Checklist.docx*](http://journals.plos.org/plosone/s/file?id=dc11/PLOSOne_Human_Subjects_Research_Checklist.docx) *(Contact us at* [*plosone@plos.org*](mailto:plosone@plos.org) *if you cannot access the document.) There may be overlap between the checklist items and other queries listed below; please address any duplicated queries both in your response email and on the checklist itself. Upload the completed Human Participants Research Checklist as file type “Other” when you re-submit your manuscript.*

We have uploaded the completed form with this resubmission. As well, as requested in the document, we have uploaded the original approval documents for all three studies.

Although the month and year for the recruitment period of each study was included in the manuscript, we neglected to include the specific days. This information has been added to the revised manuscript and can be found on pages 17-18.

Best regards,

Andrew Dawson
