# Supplementary material for: Is trust a zero-sum game? What happens when institutional sources get it wrong
Source: PLoS One. 2025 Apr 22;20(4):e0321743. doi: 10.1371/journal.pone.0321743 (PMC12013948; doi:10.1371/journal.pone.0321743)
Supplement: Attachment — Submitted filename: Response To Reviewers November 2024.docx [file pone.0321743.s004.docx]

Dear Dr. Barclay and anonymous reviewers,

Thank you for your comments. We appreciate your in-depth and thoughtful assessment of our paper. We have done our best to thoroughly address each of your comments both in the response below and in the revised manuscript. Overall we believe that the manuscript has been significantly improved by these changes based on your feedback.

We include all editor and reviewer comments below (italicized) followed by our response to each point (non-italicized).

**Editor (Dr. Barclay)**

*Regarding Reviewer 1’s point about the lack of post hoc tests, please have something that gives the specific details of your comparisons. I see that Tables 3-5 & 9 use subscripts to denote which conditions are statistically significant – this is good. However, in the interest of Open Science, it would be good to have more details on these comparisons, such as the effect sizes of the differences, or at least the value of the contrast or post hoc test. (Right now we don’t even know what statistic you used to infer these statistically significant effects.) This information should be put in Supplementary so as to not clog up the main text, except for the nature of the post hoc test (e.g., Tukey, Scheffe) which should be mentioned in the main text.*

We agree that the post hoc tests were not presented as clearly and comprehensibly as they could have been. To clarify that we ran Fisher’s LSD post hoc tests for all of our comparisons with more than two groups, we have added a sentence to the first paragraph under the “Main Analyses” heading. We have also added Tables S11-S17 in the supporting information document to provide exact t- and p-values, with an additional sentence in the note at the end of Tables 3-9 directing readers to this supplementary info.

For additional information, we have also added Figure S1 to provide the Cohen’s d values for key comparisons (namely those between the control condition and all other conditions). This is also referred to in the note for Table 7. We decided to focus on these key comparisons due to the large number of analyses and the fact that these comparisons are what are most relevant to our hypotheses (all hypotheses are written in terms of comparisons between the control condition and other conditions).

*Please note that I don’t necessarily agree with Reviewer 1’s suggestion about presenting means in text instead of the tables – you have many means and ANOVAs, so in my opinion it makes sense to have them as tables for easy reference. However, do consider whether it makes sense to have something in the text – this is especially important for summarizing an effect that is found (or not!) in all three studies, because otherwise that currently requires that readers cross-reference three different tables. Alternately, the three studies could be combined into a single table: in each section (e.g., Source), you could have different lines for each of Study 1, 2, and 3. That would make it easier for readers to see which effects replicate across studies and which effects do not. This comment means that I also don’t necessarily agree with Reviewer 1’s recommendation to present each study separately, as it might actually be easier to present the even more together in one table. However, you should still seriously consider these recommendations and either implement them, or if you think it would weaken the paper to do so, then explain your rationale for not implementing them.*

This is something we gave great consideration, as we agree with both of your concerns regarding the lack of flow and the difficulty in flipping between the main text and tables.

In the end we decided on your suggestion to combine Tables 3-5 together in order to easily compare across studies. However, we were concerned that having them all in a single table would be overwhelming and would make it difficult for readers to use it as a reference. For additional clarity, we decided to divide the tables by main effect and interaction. That means that the results of the main analyses are broken down in a simplified way: For each main effect and interaction (in order of increasing complexity), we have a section in the text, immediately accompanied by a short table showing the results for only that particular main effect/interaction across each study. The order is as follows:

- A paragraph on the main effect of source, followed by Table 3
- A paragraph on the main effect of political party, followed by Table 4
- A paragraph on the main effect of error condition, followed by Table 5
- A section on the source × political party interaction (testing Hypotheses 1a-1c), followed by Table 6
- A section on the source × error condition interaction (testing Hypotheses 2a-4b) followed by Table 7
- A section on the political party × error condition interaction, followed by Table 9
- A section on the three-way interactions, followed by Table 10

Throughout, we have added additional signposting to clarify that 1) all of these main effects and interactions are part of the same mixed ANOVA, and 2) our hypotheses are focused on the source × political party and source × error condition interactions.

We believe that having smaller tables easily accessible directly after the relevant sections/paragraphs also means that readers can easily reference the exact means and inferential statistics without requiring any additional numbers in-text.

Note that we have opted to keep the tables on perceptions of political influence (now Tables 11 & 12) in their previous configuration, since the methods are slightly different between Studies 2 and 3 (univariate ANOVA in S2 vs mixed ANOVA in S3) and the results are not broken down in as much detail in general.

We have also combined all of the correlation tables into one (now Table 11) for simplicity as well.

*Definitely add error bars to your figures, and specify in the title/caption whether the error bars are SEM, SD, or 95% CI. Error bars are a requirement nowadays at most journals so that readers get a sense of the distribution.*

We have updated both figures to include error bars and indicated in the figure captions that they refer to standard errors. We have additionally added color to the figures for clearer reading.

*Make sure that your data are publicly available, they’re currently not because it requires permission. You can create an anonymous version of the OSF page, or make your page not require permission to view (make it public after acceptance).*

Apologies for the oversight. The project is now set to public, and we have verified with colleagues that it can be accessed by accounts other than our own.

Because the OSF was not visible to reviewers previously, we do want to call attention to one item in the OSF that could prompt concern without adequate explanation. Note that although the paper reports Studies 1-3, the page on OSF contains a preregistration for a planned Study 4. This was a study that we did collect data for as part of the first author’s dissertation. However, due to an error in the survey coding, random assignment was incorrect for a number of conditions (some people received two conditions’ manipulations, and others received neither manipulation). This rendered the data set invalid as a test of the core hypotheses it was designed to assess. Because of resource constraints (limited budget to run another study) and time considerations, we opted not to run the study again, especially since we and the members of Dr. Dawson’s dissertation committee all felt that the Study 1-3 package stood well on their own as an initial set of test of the competing models and as a complete dissertation package.

We are fully transparent about the existing of the additional study listed in OSF which was rendered invalid by our coding error. We even attempted to conduct exploratory analyses on a few of the conditions that were not affected by the coding error and report that in OSF. Although findings from the salvaged conditions were consistent with Studies 1-3 we judged the incomplete, unbalanced study design to be unable to offer a clear enough test of any hypothesis to include in the manuscript.

For the sake of transparency, under the “Study 4 Planned” folder on the main page of the project, we include a document labelled “READ FIRST Planned Study 4” that gives an overview of the design of Study 4 and the results of the mean comparisons across the experimental conditions that still had useable data. We do not believe that the remaining experimental conditions are sufficient to answer our research questions or to stand alone in the paper, but we do report them in the document on OSF and find that nothing contradicts the results of Studies 1-3.

Because the coding error rendered the planned Study 4 design invalid as a test of hypotheses, we do not make reference to it in the main paper (the error takes some space to explain, and it seemed to be a lengthy tangent of little informational value if added to the main text). We provide the Study 4 transparent documentation in OSF, and we have also flagged this study in the supplementary materials under divergences from preregistration. However, if you believe that more information is warranted, we would be happy to add something to the manuscript in order to clarify further.

*The results are a little long – they could be trimmed to focus on main findings, and less important results could be relegated to Supplementary (and only referred to in main text).*

In rearranging the results section as described above, we focused our efforts on making better use of the space and making everything easier to read, though that didn’t always shorten the document.

While the sections on the correlations, additional sources, and perceptions of politicization may seem less central than the main tests of the hypotheses, we believe they are important for contextualizing and interpreting the results, so most of them have been left in. We did additionally remove a paragraph from the Perceptions of Politicization section, however, in hopes of keeping it more concise.

*If the patterns are different in different studies, I strongly recommend an internal meta-analysis (i.e., a meta-analysis of all three studies in the manuscript). This would smooth any chance fluctuations in the effect size or significance, and would allow you to draw a stronger conclusions.*

We have added an internal meta-analysis for the results of our manipulation on each trust measure.

For ease of interpretation, we focused only on the key comparisons that would directly test the predictions made in Hypotheses 2a-4b, namely the comparisons between the control condition and each of the error conditions. We used the raw means and standard deviations to produce Cohen’s *d* values, which were then put into a random-effects model using the Hunter-Schmidt estimator (Hunter & Schmidt, 1990). A separate meta-analysis was run for each comparison and each trust measure (mainstream trust, counter-mainstream trust, neutral trust), producing nine overall estimates in total.

The results are shown in what is now Table 8 in the manuscript, with the preceding two paragraphs describing the process and results. To keep the existing flow of the results section, we included the internal meta-analyses in the same section immediately following the original source × error condition results, as these are the results we were trying to aggregate across studies.

Overall, the results strengthen the support for the General Loss Model, with all effects significant with the one exception of the comparison between the control and neutral error conditions for the mainstream source. We believe this demonstrates considerable consistency in our findings across studies.

Minor comments:

*Table 1 might be easier if it has up & down errors & “no change” for each condition*

We were not quite sure what was requested here, but gathered that it was a call for greater clarity in presenting the results, which we have aimed to do in several ways (reorganizing text and tables for mixed ANOVAs, improvements to the figures, adding details on multiple comparisons in the supporting info).

*Tables 3 & 4 might be easier to understand if rearranged or made into more graphs*

See response above on how we rearranged Tables 3-5 into multiple smaller tables, in which main effects and interactions are broken down across studies immediately after they are discussed in-text. We believe this new arrangement improves the clarity and flow of the paper.

*In Table 3, for the Source x Error interaction, Neutral row, shouldn’t the Counter-Mainstream column have the subscript a instead of b? In general, double check all subscripts*

Good catch, you are correct. We have corrected the subscript. We have also doubled-checked all of the subscripts across tables and verified that they are correct. The only additional fixes needed were to change one subscript in Table 10 and placing the subscripts in the Error Condition × Political Party section in Table 9, which were missing originally.

**Reviewer 1**

*The authors report the results of ANOVAs but do not specify whether post hoc tests were conducted to examine group differences. This omission makes it difficult to assess the robustness of the conclusions. Without post hoc analyses (or planned contrasts), it is unclear whether the group differences are statistically significant. The authors should either conduct or clearly report post hoc comparisons to demonstrate that the effects are meaningful. Additionally, improving the organization of the results section would enhance clarity and ease of interpretation.*

As indicated above, we agree that the post hoc tests were not presented as clearly and comprehensibly as they could have been. To clarify that we ran Fisher’s LSD post hoc tests for all of our comparisons with more than two groups, we have added a sentence to the first paragraph under the “Main Analyses” heading. We have also added Tables S11-S17 in the supporting information document to provide exact t- and p-values, with an additional sentence in the note at the end of Tables 3-9 directing readers to this supplementary info.

*Organization:
• Since the introduction is long, include a brief summary at the beginning to highlight the research question/problem, going straight to the point and outlining what readers should expect from your introduction.*

*• Consider presenting the relevance of the paper more directly. For instance, start with the core issue (e.g., lack of trust in certain institutions) before discussing the consequences of this distrust.*

While we have not drastically changed the introduction section, we have restructured the beginning to get to the point a little more efficiently. The first paragraph focuses on the case of COVID-19 as a hook, the second paragraph discusses the issue of declining trust across institutions, and the third paragraph describes our research questions more explicitly.

*• Avoid short paragraphs and consider merging them (e.g., the first paragraph of the paper).*

This is a stylistic preference, so we have tried to remove short paragraphs where they were unnecessary, but kept them in cases where we think they add to clarity. The first and second paragraphs in the paper have been made into one, as have the two paragraphs under the heading of “General Loss Model” in the discussion. However, we decided to stick with short paragraphs when summarizing each study at the end of the introduction, as well as summarizing each source (mainstream, counter-mainstream, neutral).

*Language Precision:
• Use more precise language (e.g., avoid vague terms like "several"). If possible, quantify or specify where appropriate.*

We have changed to more precise language wherever specific numbers could be given, namely in two main areas: We clearly state throughout that there are four models that we are testing, and where we previously stated that trust was declining for “several decades” we now state “since at least the 1970s” since that is what is indicated by the articles we cited.

*• Ensure that claims are backed by references or examples (e.g., on page 5, the first line of the second paragraph requires examples or sources).*

In this particular example, we intended to refer to examples in the subsequent sentences to build on this general point. However, because it is quite a general statement, we have softened the language slightly to avoiding coming across as accusing researchers in this area from altogether ignoring potential connections between sources.

*• In sections discussing trust in institutions, include specific numbers to contextualize the severity of the problem.*

Exact numbers are difficult to narrow down on without getting too far into the weeds, as there is considerable variation in the questions asked, the specific institutions considered, and the subgroups showing change. However, for some sense of context, we did add one line when the issue is introduced: “For instance, the proportion of Americans reporting a “great deal” of trust in medicine declined by about 30% [since the 1970s]”.

*Materials and Methods:
• Consider moving descriptions of the stories used in the studies from "Current Research" to "Materials" and minimize methodological details in the introduction.*

These three paragraphs have been moved from the end of the introduction to the beginning of the materials section. We agree that this placement makes more sense given their methodological focus.

*• Ensure the "Procedures" section focuses on what participants did during the study. Avoid repeating information already covered in "Materials."*

We believe that the source of overlap/repetition you’re referring to is in how we describe the different scenarios used in each study at the end of the introduction, just before the Materials and methods section begins, as well as in the procedure section. We have removed this discussion from the introduction, so that the different scenarios are only described once (in the procedure section). This change should cut down on any redundancies.

We have included a paragraph at the beginning of the Materials and methods section just to explain how the studies are reported together and that the data and preregistrations are available on OSF.

*• Clarify the sequence of events (e.g., whether participants completed demographic questions before or after the manipulation) to improve clarity for replication. Present a step-by-step of what participants did.*

We have added phrasing throughout to clarify that the order was Demographics, Manipulation, Primary Trust Measures, Additional Trust Measures, Political Influence Measures, and Memory checks.

*Study Presentation:
• Consider reporting each study separately to improve clarity and narrative flow. Present the methods, results, and discussion for Study 1 first. After that, when introducing Study 2 (and later Study 3), emphasize both the similarities and differences between the studies. For example, you could say: "The procedures in Study 2 follow those of Study 1, except that in Study 1 we did X, whereas here we are doing Y." This approach helps maintain continuity while clarifying how each study builds on the previous one. Currently, the presentation is confusing due to the numerous caveats associated with each study.*

*• Consider reporting the results for each study separately (Study 1: Methods, Results, Discussion; Study 2: Methods, Results, Discussion, etc.).*

*• Consider organizing the results in a manner that tells a story. Rename the headings to reflect either the research questions or the key findings, depending on which approach better conveys a cohesive narrative.*

In line with the editor’s suggestions for this issue, we have opted to keep all three studies together but make additional changes to improve clarity and flow. Most importantly, the main results have been rearranged to more clearly break down the main effects and interactions. Each table presents one main effect or one interaction, comparing studies side-by-side. We have also added additional signposting to clarify when we are testing the main hypotheses. We believe this will improve the sense of narrative by clearly guiding readers through each step of the analysis process while still providing focus on the analyses that test our main hypotheses.

Several changes have been made to the method section as well, to clarify the order of the procedures and measures and remove redundancies when describing differences between studies.

*• Move all measures descriptions (e.g., primary and additional trust measures) into the "Materials" section and do not give any procedures description under "Materials".*

The measure descriptions should all be located in the Materials and methods section under the Primary Trust Measures, Additional Trust Measures, and Political Influence Measures headings. As noted above, we have removed the descriptions of the different scenarios from the end of the introduction section so that the scenarios are only described in the procedure.

*• Memory checks do not need to be described in detail. Memory, attention, and manipulation checks are typically mentioned under "Participants" for the whole purpose of informing about exclusions. If they are relevant to your research question, then provide an explanation.*

Although we recognize that basic attention checks often appear just as exclusions, our memory checks are reported to show how clearly people understood the information in the manipulations, which can help readers assess the strength of the manipulation and the results. We tried to keep this section as concise as possible, but believe that it should be left in.

*Results and discussion:
• Right after the main analyses heading (paragraph 1), clearly describe the levels of each variable (e.g., error condition, political party) so that readers do not need to refer back to the "Methods" section.*

Good idea to improve clarity. We have added levels in parentheses for both variables.

*• Clearly spell out the results in the text. While you do this in some sections, other parts are overly technical. Avoid relying too heavily on tables for key findings. Bring in the means and standard errors (SE) directly into the narrative and avoid unnecessary statistical jargon.*

*• Consider moving the ANOVA tables to supplementary materials, as tables should provide additional details rather than being the primary source of the findings.*

As discussed above, we have opted to keep most of statistics in the tables, but have rearranged the tables so that the relevant table accompanies the section of text in which it is referenced. We believe this improves narrative flow and helps with comparison while keeping statistical jargon out of the text.

*• Ensure that graphs include error bars to visually convey variability and precision.*

We have updated both figures to include error bars and indicated in the figure captions that they refer to standard errors.

*When you state, "The neutral source was also trusted more than the counter-mainstream source in Studies 1 and 3," did you conduct post hoc tests or planned contrasts to specifically compare between groups? It appears that only ANOVAs were performed, and once you find a significant ANOVA, you just look at the means and infer that group A is greater than group B. However, ANOVAs alone do not indicate which groups differ from one another. Post hoc tests (or planned contrasts) are necessary to draw meaningful conclusions about group comparisons. If the ANOVA results are significant, it only tells you that at least one group mean is different from the others, but it does not reveal which groups differ. ANOVA does not break down these comparisons; it only assesses the overall variance among the groups. For instance, if you have three groups (A, B, and C) and ANOVA finds a significant difference, it doesn't indicate whether A differs from B, A differs from C, or B differs from C. You need post hoc tests for making these sorts of claims. Include these tests to support your interpretations for every group comparison made.*

Post hoc tests were performed for all ANOVAs, using Fisher’s LSD. The differences between each cell were indicated using subscripts. However, as indicated above, we agree that this was not communicated as clearly as it could have been.

To clarify that we ran Fisher’s LSD post hoc tests for all of our comparisons with more than two groups, we have added a sentence to the first paragraph under the “Main Analyses” heading. We have also added Tables S11-S17 in the supporting information document to provide exact t- and p-values, with an additional sentence in the note at the end of Tables 3-9 directing readers to this supplementary info.

*I could not access the OSF for this project because it required permission.*

Apologies for the oversight. The project should now be public and accessible through the same link.

**Reviewer 2**

*Unfortunately, I was not able to access the OSF documents with the preregistrations so I didn’t have the chance of comparing what was preregistered with what was done, though this is also mentioned in the supplementary materials, so it didn’t affect my review. (However, this also prevented me from checking whether the data was uploaded. As a note for the future, the authors could make use of the view-only (anonymous) link creation possibility on OSF to provide accessible links during the review process.)*

Apologies for the oversight. The project should now be public and accessible through the same link.

*On another technical point, I would suggest adding confidence intervals to the graphs in Figures 1 and 2.*

We agree with this suggestion and have added error bars to both graphs. However, we have opted to use standard errors, for the sake of consistency with the tables.

*I was slightly confused with the social alignment model and the rationale behind it. I quote from the ms., “when one source loses trust, only sources that are framed directly in opposition to that source (e.g. counter-mainstream against the mainstream source) will gain trust, while neutral, unaligned sources will not.” I’m not sure I understand this premise, especially when it concerns losing trust in a single counter-mainstream source. I would think that people can just pick another one, given their apparent number in the current informational environment? I would have thought social alignment to have a different effect – that of shifting trust to another counter-mainstream source which provides the individual with the same type of information, but hasn’t shown to have made a large error and thus lost its credibility. Unfortunately, there was no "other from the same category" type in the design.*

*My general point being, it might be more likely to find zero-sum logic of trust between sources in the same category rather competing ones. We don’t only use information to make decisions that have straightforward consequences on our well-being -- for a lot of the information we do have, it isn’t necessarily strictly important that it’s right as much as that our group might believe it/trust the source. From this view, there could have been another model which would predict that people could increase trust for other similar sources which haven’t lost their credibility. This comment relates to, in part, to Dan Williams’ work on rationalization markets. As far as I understood the design, there wouldn’t be a clear way to tease that apart from the studies as are.*

Your point touches on an underlying assumption of the Social Alignment Model that we failed to communicate when we introduced it in the original draft of the paper. The model assumes that “mainstream” and “counter-mainstream” respectively are viewed as collectives, two aggregate groups in competition with each other. Therefore in this model, if one mainstream source lost trust, we would not expect people to shift to another mainstream source, as they are part of the same “side” rather than viable alternatives to each other. People in this model are then only shifting between the two “sides”, leaning more towards one or the other at a given time. We have updated the description of the Social Alignment Model in the introduction to make this assumption more explicit.

This leaves open the question of whether people could in fact shift between sources on the same side (e.g., from one mainstream source to another). While you are right that this possibility was not built into our main analyses, it was addressed in part by our analyses of “Additional Trust Measures”. In that section we compare trust in a variety of different sources across conditions, and find no evidence that any of the additional mainstream sources gained trust when the primary mainstream source lost trust (and same for counter-mainstream sources). We have added a few sentences in this section to tie this into the assumption of the Social Alignment Model, where all sources on each side are tied together. We also note as a future direction in the discussion that future studies could include multiple mainstream and/or counter-mainstream sources to better assess whether people are inclined to switch between options within one “side”.

Note that it is also possible that people may be inclined to switch to a different source on the same *political* side as themselves. This concern is addressed in Study 3, where the political leaning of all sources were held constant on the same side as the participant.

*Finally, it isn’t entirely clear from the vignettes how the error correction came about. For example, if it was an intentional error (going against the grain to reach a certain audience) vs. an “honest” mistake which came from lack of initial information, or some other unintentional cause. I think people might view the decision making process behind publishing information by the FDA differently than a media outlet which subsists on clicks. I wonder whether there are different expectations in terms of accuracy tied to the different organizations, which could also affect this dynamic? The gravity of the errors might not be perceived the same either, especially since people probably don’t use e.g. a sports site to learn information on which to make decisions about their health. I think the authors should probably discuss this distinction somewhere in the manuscript.*

The motivation behind the error was intentionally kept ambiguous, in order to avoid bringing any additional factors into play and to align with real-world scenarios where the source’s motivations are up for interpretation. We have added a sentence to our description of the manipulation (under the “Manipulation” heading).

However, you are right that this ambiguity can be a limitation as much as a feature, since the results might depend on people’s interpretation of the motivations. To acknowledge this, we have added a sentence under the “Politicization” heading in the general discussion: “This highlights a key limitation in our manipulation: the intentions of the source were left largely ambiguous, focusing on the error itself rather than any potential political bias.”

**Journal Requirements**

*1. Please ensure that your manuscript meets PLOS ONE's style requirements, including those for file naming. The PLOS ONE style templates can be found at*

[*https://journals.plos.org/plosone/s/file?id=wjVg/PLOSOne_formatting_sample_main_body.pdf*](https://journals.plos.org/plosone/s/file?id=wjVg/PLOSOne_formatting_sample_main_body.pdf) *and*

[*https://journals.plos.org/plosone/s/file?id=ba62/PLOSOne_formatting_sample_title_authors_affiliations.pdf*](https://journals.plos.org/plosone/s/file?id=ba62/PLOSOne_formatting_sample_title_authors_affiliations.pdf)

We have added line numbering throughout the document, which we realized had been missing after the abstract. Otherwise, we have followed PLOS One style guidelines to the best of our knowledge. Please advise if there are any other specific guidelines we have overlooked.

*2. Please provide additional details regarding participant consent. In the ethics statement in the Methods and online submission information, please ensure that you have specified (1) whether consent was informed and (2) what type you obtained (for instance, written or verbal, and if verbal, how it was documented and witnessed).*

We have updated the method section to state that consent was informed and written.

*3. Please include captions for your Supporting Information files at the end of your manuscript, and update any in-text citations to match accordingly. Please see our Supporting Information guidelines for more information:* [*http://journals.plos.org/plosone/s/supporting-information*](http://journals.plos.org/plosone/s/supporting-information)*.*

As we understand it, the supporting information listed at the end of the manuscript in the formatting guidelines refer to additional figures and tables that will still be part of the main paper. This is different from the separate document we have for our supporting information, which is formatted like a separate paper which discusses additional analyses in-depth with its own figures and tables. Please advise if this needs to be changed.

We would like to thank the editor and review team once again for their detailed and thoughtful analysis of our manuscript, which we believe has made it into a much stronger paper. We hope that our responses above demonstrate that we have carefully considered each comment and done our best to address your concerns.

Thank you for your valuable time and for the opportunity to be considered for publication in PLOS One.
