# Supplementary material for: Is trust a zero-sum game? What happens when institutional sources get it wrong
Source: PLoS One. 2025 Apr 22;20(4):e0321743. doi: 10.1371/journal.pone.0321743 (PMC12013948; doi:10.1371/journal.pone.0321743)
Supplement: Attachment — Submitted filename: PlosOne Revision 2.docx [file pone.0321743.s003.docx]

Thank you for addressing my previous concerns. The manuscript is much clearer now, and I was able to access the OSF page without any issues.

The methods and procedures are also much clearer in this revision, which strengthens the overall presentation. I also found the results section to be cohesive and easy to follow, which enhances the clarity of the findings.

Additionally, while you clearly explain that the hypotheses related to the general loss model were not pre-registered, this distinction should be explicitly marked in the hypothesis table itself. Doing so will ensure readers are immediately aware (or reminded) of which hypotheses were pre-registered, and which were not, helping to maintain clarity throughout.

Also, I have just one minor suggestion regarding the numbering of hypotheses. I understood what they referred to after reading the captions in your hypothesis table. However, the numbering can be a bit confusing at first glance—especially since seeing 1, 2, and 3 naturally leads readers to associate them with the studies. This might be something to consider for added clarity.

Aside from these minor points, I have no further suggestions. I believe the paper is in good shape for publication.

Best regards, Reviewer 1.
